# An autonomous fabric electrochemical biosensor for efficient health monitoring

**DOI:** 10.1093/nsr/nwaf155

**Published:** 2025-04-23

**Authors:** Liangliang Zhou, Changxin Li, Yongfeng Luo, Qimin Liang, Yangyang Chen, Zhuojun Yan, Longbin Qiu, Sisi He

**Affiliations:** Hunan Province Key Laboratory of Materials Surface & Interface Science and Technology, College of Electronic Information and Physics, Central South University of Forestry and Technology, Changsha 410004, China; Shenzhen Key Laboratory of Flexible Printed Electronics Technology, School of Science, Harbin Institute of Technology (Shenzhen), Shenzhen 518055, China; Shenzhen Key Laboratory of Flexible Printed Electronics Technology, School of Science, Harbin Institute of Technology (Shenzhen), Shenzhen 518055, China; Hunan Province Key Laboratory of Materials Surface & Interface Science and Technology, College of Electronic Information and Physics, Central South University of Forestry and Technology, Changsha 410004, China; Shenzhen Key Laboratory of Flexible Printed Electronics Technology, School of Science, Harbin Institute of Technology (Shenzhen), Shenzhen 518055, China; Shenzhen Key Laboratory of Flexible Printed Electronics Technology, School of Science, Harbin Institute of Technology (Shenzhen), Shenzhen 518055, China; Shenzhen Key Laboratory of Flexible Printed Electronics Technology, School of Science, Harbin Institute of Technology (Shenzhen), Shenzhen 518055, China; Department of Mechanical and Energy Engineering, SUSTech Energy Institute for Carbon Neutrality, Southern University of Science and Technology, Shenzhen 518055, China; Shenzhen Key Laboratory of Flexible Printed Electronics Technology, School of Science, Harbin Institute of Technology (Shenzhen), Shenzhen 518055, China

**Keywords:** fabric biosensor, fiber-shaped device, health monitoring, sweat induction, wearable

## Abstract

As a promising frontier in next-generation healthcare monitoring, smart textiles that are capable of dynamic physiological sensing through sweat analysis represent an emerging paradigm in wearable electronics. However, the inherent inaccessibility of sweat in sedentary individuals and scenarios has restricted our ability to capitalize on this non-invasive and insightful source of molecule-level information. Here, we first present a comfortable, autonomous and integrated iontophoretic biosensing textile system that features an on-demand stimulation skin-interfaced sweat-induction unit. The textile system uses skin-interface stabilized iontophoretic hydrogel electrodes that enhance interface conformability and optimize interface impedance, eliminating the need for high-current stimulation in conventional iontophoresis. By combining biosensing fibers with a stabilized transduction layer design, we show that the resultant biosensing textile system continuously collects multibiomarker data, including glucose, lactate, uric acid and pH levels, for up to 6 hours. This system holds promise for advancing wearable electronics in personalized healthcare, clinical monitoring and remote diagnostics with superior user-friendliness and versatility.

## Teaser

An autonomous fabric biosensor integrating skin-interface stabilized iontophoretic hydrogel electrodes with robust biosensing fibers enables autonomous sweat induction and long-term multiplex biomarker monitoring, paving the way for non-invasive and scenario-independent personalized healthcare.

## INTRODUCTION

Recent breakthroughs in wearable electronics are revolutionizing the face of personalized healthcare and telemedicine, moving beyond traditional approaches that require invasive routine check-ups or symptom-triggered interventions [1–4]. As a promising contender for next-generation wearable electronics, smart textiles have attracted significant research attention due to their softness, permeability, durability and comfort [5–7]. Prolific research efforts have been dedicated to wearable fabric electronics, branching out from tracking biophysical signals during physical activity to monitoring biochemical signals in biofluids [8–11]. This evolution provides more insightful physiological information at the molecular level, progressively blurring the boundary between users and wearable healthcare devices.

Sweat is an attractive biofluid that is valued for its wide accessibility, abundant biochemical molecular information and, more importantly, for being non-invasive and enabling continuous monitoring [12–14]. Recent advances in biosensing fabrics have been successfully applied to real-time biosignal monitoring in sweat, including detecting electrolytes, metabolites, nutrients and so on [15–18]. Efficient sweat sampling marks the initial step toward reliable biomarker analysis for sweat-based sensors. Nonetheless, the inherent inaccessibility of sweat in sedentary individuals and scenarios has restricted our ability to capitalize on this non-invasive and insightful source of molecule-level information. Although various strategies involving porous hydrogels and hydrophilic fillers have been explored [19–21], obtaining sufficient and reliable biofluid for sensor analysis remains challenging due to low secretion rates and sample dilution. The confined applicability to specific user groups and scenarios, combined with low secretion rates and sample dilution, limits the potential for widespread adoption. Alternatively, iontophoresis achieves active sweat induction through epidermal electrical stimulation to overcome the limitations regarding specific scenarios and target populations, but raises concerns about pain, burn and sweat component distortion caused by applying a high level of current stimulation [22–24]. Besides, current biosensing fabrics suffer from limited stability, which hinders their ability to continuously monitor health through sweat [25,26]. As a result, developing an efficient, comfortable and long-term health-monitoring system by using fabric-based electrochemical biosensors is crucial but challenging.

**Figure 1.**
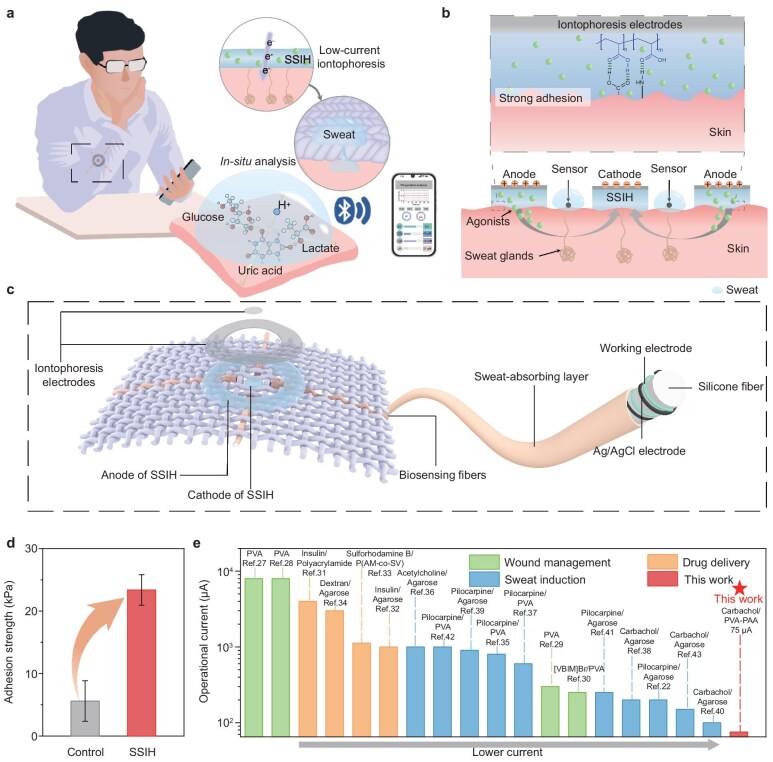
Schematics of the autonomous stimulation integrated fabric electrochemical biosensor. (a) Illustration of the fabric biosensing system and main functionalities: low-current iontophoresis sweat induction and sampling, *in situ* physiological signal analysis and wireless data transmission and interaction. (b) Schematic illustration of localized sweat sampling based on iontophoresis of the secretory agonist (carbachol) at the electrode–skin interface. (c) Schematic illustration of the electrochemical biosensing fabric system integrated with an iontophoresis unit and a biosensing fiber array. (d) Comparison of adhesion strength between control and SSIH (*n* = 3, mean ± SD). (e) Operational current of this work in comparison with previously reported iontophoresis-based transdermal healthcare systems. Data are compiled from literature and can be found in Table S1. PAN, polyacrylonitrile; PVA, polyvinyl alcohol.

Herein, we first design a comfortable iontophoretic electrochemical fabric biosensor that achieves autonomous stimulation of sweat induction and enables continuous long-term sweat monitoring. The electrochemical biosensing fabric was fabricated by combining an iontophoresis unit with a multisensing electrochemical fabric (Fig. 1a–c). Enabled by skin-interface stabilized iontophoretic hydrogel (SSIH) with superior mechanical compliance and low-impedance interface (Fig. 1a and d), the iontophoresis unit achieves conformal skin contact and transdermal activation of sweat glands under an external low operating current of 75 µA (corresponding to a current density of 32.12 µA/cm²) (Fig. 1e and Table S1) [22,27–43]. Upon contacting the sweat, the electrochemical fabric composed of multiple biosensing fibers detected corresponding biomarkers (glucose, lactate, uric acid and pH) during long-term operation for up to 6 hours, attributed to the employment of an inert protective layer to counteract the degradation of the transducer layer. As a proof of concept, we integrated the autonomous electrochemical biosensing fabric into daily wear, coupled with an *in situ* signal-processing unit and a wireless bidirectional communication module interacting with a mobile interface.

## RESULTS AND DISCUSSION

### Design of the fabric system for sweat sampling and analysis

The wearable electrochemical fabric sensing system integrates a skin-interfaced iontophoresis unit and a biosensing fiber array into a textile form, enabling autonomous sweat sampling and real-time analysis (Fig. 1c). The iontophoresis unit consists of a pair of SSIH electrodes, with an anode containing the secretory agonist (carbachol) and a complementary cathode. Assisted by an externally applied programmable current source, the agonist molecules can be delivered beneath the local skin gently and imperceptibly to stimulate local sweat glands for efficient sweat induction (Fig. 1b). The system features a cross-shaped biosensing fiber array consisting of four biosensing fibers detecting glucose, lactate, uric acid and pH, respectively. Each biosensing fiber is formed by coaxially twisting a functionalized carbon nanotube fiber (CNT)-based working electrode and an Ag/AgCl electrode around a stretchable silicone fiber, ensuring adaptability to mechanical deformation (Fig. S1). Specifically, the enzyme-based working electrode was initially fabricated by electrodepositing Prussian blue (PB), followed by the electrodeposition of nickel hexacyanoferrate (NiHCF). The corresponding enzyme was immobilized within a CNT/chitosan composite matrix, forming the active layer for selective detection. Similarly, the pH-sensing electrode was obtained by electrodepositing polyaniline onto the CNT fiber, leveraging its surface protonation changes for potential variation across different pH values (Figs S2 and S3). Meanwhile, the reference/counter electrode was prepared by dip-coating the CNT fiber with commercial Ag/AgCl ink (Fig. S4). Moreover, each biosensing fiber features an outer shell of superhydrophilic polyacrylonitrile/SiO_2_ composite nanofibers that serve as a sweat-absorbing layer. The superhydrophilic channels of the sweat-absorbing layer drive rapid sweat absorption and accumulation for downstream biosensing (Figs S5 and S6). Finally, the compact semi-punched design enables the integration of the sweat-induction unit and the biosensing fiber array, ensuring consistency in subsequent applications.

### Sweat-induction unit enabled by SSIH

Conventional iontophoretic sweat induction employs a relatively high electrical current or voltage for the transdermal delivery of cholinergic agents, activating internal and proximal sweat glands [36,44]. To mitigate potential risks from intense external stimulation, such as pain, the skin electrode is required to maintain low interfacial impedance coupled with efficient drug-delivery capabilities [41]. To this end, we developed SSIH electrodes with an adhesion-enhanced interface design, featuring a double-network architecture with superior mechanical compliance (Fig. S7). The iontophoretic electrodes were prepared by interpenetrating polyvinyl alcohol crystalline domains with poly(acrylic acid) networks. The muscarinic agent carbachol was simultaneously and uniformly loaded into the matrix, allowing autonomous delivery to the dermal space and inducing sweating. Finally, a pair of CNT films were covered on the surface to obtain the SSIH electrodes.

**Figure 2.**
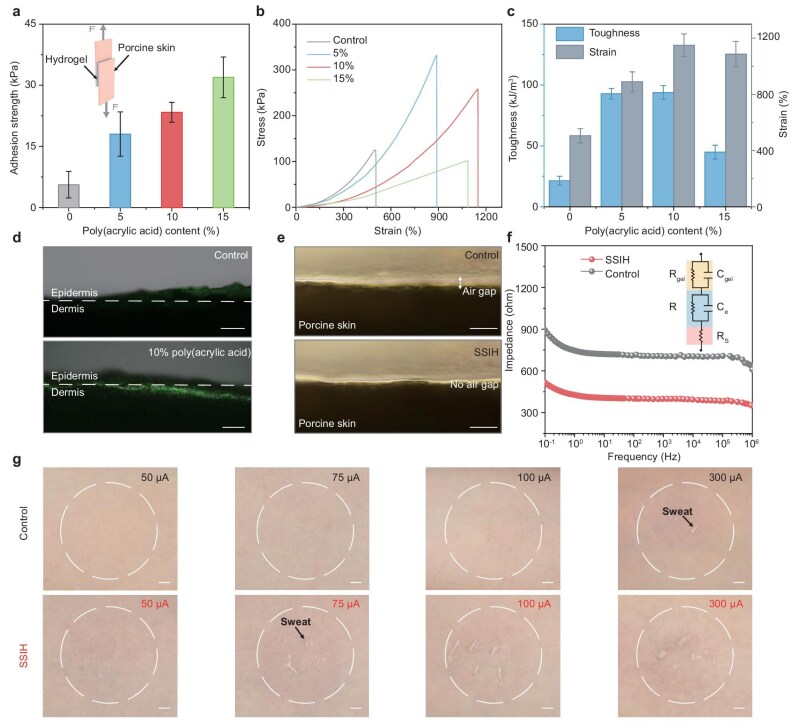
Optimization and characterization of the sweat-induction unit enabled by SSIH. (a) Adhesion strength of SSIH with different poly(acrylic acid) contents under lap-shear testing on porcine skin (*n* = 3, mean ± SD). (b) Tensile stress–strain curves of SSIH with different poly(acrylic acid) contents. (c) Toughness and strain of SSIH with different poly(acrylic acid) contents (*n* = 3, mean ± SD). (d) Cross-sectional fluorescent images of porcine skin via SSIH with 0% and 10% poly(acrylic acid) contents after a 10-minute iontophoresis process. Rhodamine B was used as a model drug for visualization; scale bar, 500 µm. (e) Cross-sectional microscopy images of the control sample and SSIH mounted on porcine skin. Scale bar, 200 µm. (f) Electrochemical impedance spectroscopy analysis of control sample and SSIH interfaced with porcine skin (inset, equivalent circuit model). (g) Comparison of sweat induction by iontophoresis based on control and SSIH electrodes under varying operational currents from 50 to 300 µA. Scale bars, 2.5 mm.

The properties of SSIH were systematically investigated, including interfacial adhesion, mechanical properties and drug-delivery capability. Adhesion strength was examined by using lap-shear tests on porcine skin to simulate human skin. As shown in Fig. 2a, the adhesion strength increased with higher poly(acrylic acid) content, primarily attributed to numerous hydrogen bonds between the hydroxyl and carboxyl groups on the SSIH surface and the amino or other functional groups on the skin (Figs S8 and S9) [45,46]. Considering the crucial role of mechanical properties in the combination within wearable flexible electronics, a series of tensile tests were conducted. The results revealed a dependence of the mechanical properties of the hydrogel on the network composition, where an increase in the poly(acrylic acid) secondary network content initially enhanced both the stress tolerance and the toughness through effective load distribution. At a 10% poly(acrylic acid) concentration, the material exhibited the most favorable mechanical properties, with a toughness of 93.82 kJ/m^3^ and an elongation at break of 1150% (Fig. 2b and c). However, when the content was increased to 15%, the excessively dense network reduced the free volume, restricting the molecular chain movement and rendering the material brittle [47]. To evaluate the effect of the SSIH composition on the transdermal drug-delivery capability, Rhodamine B (a small molecule with polarity similar to carbachol) was incorporated into the hydrogel matrix to visualize the penetration process under a confocal microscopy [48]. The SSIH with 10% poly(acrylic acid) content exhibited the highest fluorescence intensity and the deepest diffusion depth in the dermal layer among all the compositions (Fig. 2d and Fig. S10). Further scanning electron microscopy revealed that the hydrogel matrix microstructure exhibited a variable trend, transitioning from compact networks to highly porous architectures and back to condensed structures with increasing poly(acrylic acid) content (Fig. S11). The superior transdermal delivery efficiency of the SSIH with 10% poly(acrylic acid) content can be attributed to the enhanced adhesion and ideal porous structure for drug loading and transport. Therefore, the poly(acrylic acid) weight ratio was fixed at 10% in the SSIH for the subsequent investigation.

The SSIH exhibited enhanced adhesion, effectively reducing the microscale non-conformal contact during skin-surface interactions compared with the control sample (without poly(acrylic acid) incorporation), as verified by the cross-sectional microscopy images (Fig. 2e). Encouragingly, after more than 20 repeated lap-shear cycles and during the 5-hour test under representative wearable conditions (25°C and 60% relative humidity) [49,50], the adhesion strength showed slight attenuation but remained at >10 kPa, while the retention rate was maintained at 90% (Fig. S12). Meanwhile, the SSIH demonstrated reliable adhesion under fluctuating environmental conditions (Fig. S13). The effect on interface impedance was further investigated through detailed impedance testing, in which the electrode–skin interface impedance was fitted by using an equivalent circuit model consisting of a hydrogel electrode, an epidermal layer and a subcutaneous layer. In this model, the SSIH electrode resistance and capacitance are denoted as *R*_gel_ and *C*_gel_, respectively. *R*_e_ and *C*_e_ represent the resistance and capacitance of the epidermal layer, while the resistance of the subcutaneous layer is denoted as *R*_s_ [33,51]. The SSIH–skin interface exhibits lower interfacial impedance across all frequency ranges than the control sample, owing to its enhanced conformal contact and superior skin compliance (Fig. 2f).

Before evaluating the feasibility of sweat induction *in vivo*, we conducted cytocompatibility testing on hepatocytes, confirming the satisfactory biocompatibility of the SSIH and its potential for skin-interfacing applications (Fig. S14). No skin irritation or allergic reactions were observed throughout the 8-hour *in vivo* wear test, comprehensively validating the safety of the SSIH for long-term human use (Fig. S15). In this case, we performed iontophoresis on the human forearm by using a programmable constant current output. The SSIH induced noticeable sweat at a lower current of 75 µA, whereas the control sample only began to produce sweat at 300 µA (Fig. 2g). To visualize the sweat droplets, we applied a water-sensitive black dye that turned blue upon contact with moisture (Fig. S16). Furthermore, we validated the system on a larger cohort of subjects to enhance the generalizability of the SSIH (Fig. S17 and Table S2). As the applied current increased, the sweat output of the SSIH-based iontophoresis unit did not increase further, which can be attributed to the limited local sweat gland density and the resistance caused by accumulated sweat on the epidermis. Moreover, the SSIH exhibited slight resistance changes over a 7-hour period, further demonstrating its stable skin–electrode interface and reliable long-term operation (Fig. S18). As a result, our skin-interfaced iontophoresis unit demonstrates competitive performance and extremely low current requirements (device driving current of 75 µA, current density of 32.12 µA/cm^2^) compared with previously reported transdermal healthcare systems based on iontophoresis, including sweat induction, drug delivery and wound management (Fig. 1e and Table S1). The lower current highlights its advantages regarding transdermal delivery efficiency and safety for developing painless and burden-free electronics.

### Electrochemical sweat-sensing performance of the fabric system

**Figure 3.**
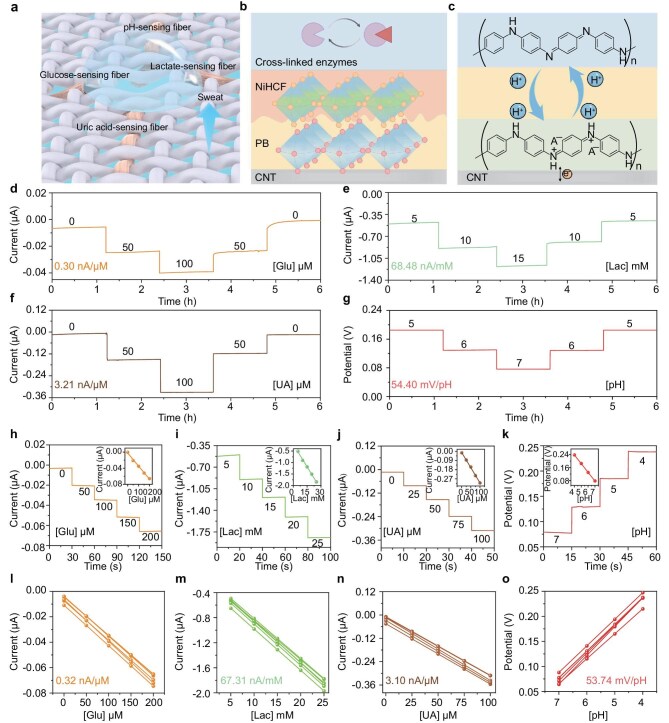
Optimization and evaluation of the electrochemical fabric for multiplexed sweat biosensing. (a) Schematic of the fabric biosensing system for sustained sweat sampling and analysis. (b and c) Configurations and mechanisms of enzymatic biosensing fibers and pH-sensing fibers. (d–g) Operational long-term stability of (d) glucose, (e) lactate, (f) uric acid and (g) pH biosensing fibers. (h–k) Current responses of (h) glucose, (i) lactate and (j) uric acid biosensing fibers and (k) open-circuit potential responses of pH biosensing fibers to the corresponding analytes in phosphate-buffered saline. Insets in (h–k) present the corresponding sensor calibration relationships; the correlation coefficients are 0.999, 0.997, 0.998 and 0.999, respectively. (l–o) Reproducibility of the (l) glucose, (m) lactate, (n) uric acid and (o) pH biosensing fibers (*n* = 6), with relative standard deviations of 7.43%, 5.63%, 7.47% and 5.61%, respectively.

Sweat metabolites (including glucose, lactate and uric acid) serve as vital biomarkers for various physiological conditions such as diabetes, metabolic state changes, inebriation and renal dysfunction [52]. In our designed electrochemical fabric biosensing system, the sweat-induction unit enabled by the SSIH permits effective sweat sampling, ensuring optimal conditions for the downstream operation of biosensing fibers (Fig. 3a). Typically, the specific enzyme, acting as a bioreceptor, is deposited on the surface of the PB/CNT electrode to construct the enzymatic metabolite biosensing fibers. The mechanism of the enzymatic biosensor is that electrolytes in sweat undergo selective oxidation, resulting in corresponding oxidized products and hydrogen peroxide (H_2_O_2_) [53]. However, the process can induce electrode polarization effects that compromise measurement signal stability [54]. As an ion-to-electron transducer layer, the PB layer facilitates redox reactions with H_2_O_2_ at near-zero potential and ensures stable signal transfer [55]. Nonetheless, a significant limitation of PB lies in its incomplete stability in biofluids, in which hydroxide ions (OH⁻) in neutral and alkaline solutions break the Fe–(CN)–Fe bonds within the PB lattice and exacerbate its dissolution. The stability evaluation of PB/CNT electrodes through repeated cyclic voltammetry scans under various pH conditions revealed a pronounced decline in PB peak intensity, demonstrating its progressive degradation in aqueous media (Fig. S19). In response, we introduced the PB-analog NiHCF on the surface of the PB transducer layer by using a sequential deposition process, as illustrated in Fig. 3b. The NiHCF exhibited superior chemical inertness, which can form a protective layer over the PB redox center. Moreover, the insertion of Ni^2+^ is expected to reduce the lattice constant of ferrocyanide Fe_4_[Fe(CN)_6_]_3_, thereby reinforcing the lattice and mitigating potential lattice distortion caused by repeated ion or water insertion when applied in sweat analysis [56,57]. After the deposition cycles were optimized, the irreversible degradation of the PB transducer layer was effectively suppressed (Figs S20–S22). Following this, the enzyme was solidified via entrapment within a chitosan network, which acts as a protective matrix, enhancing stability and preventing the efflux of the catalytic component [58]. Notably, while the inertness of nickel may be unfavorable for hydrogen peroxide transduction, the lower electronegativity of Ni^2+^ compared with Fe^3+^ induces an inductive effect that lowers the bond-force constant of the Fe–C≡N bond [59]. This reduction leads to the electrochemical activation of C-coordinated Fe ions, enhancing electrochemical activity as compensation [60]. Therefore, as the enzymatic metabolite (glucose, lactate and uric acid) biosensing fibers have a similar device architecture except for their specific enzymes, optimizing the mass loading of the composite transducer layer to obtain an optimal NiHCF/PB bilayer configuration achieves both sensitivity and long-term stability (Figs S23–S25). In addition, given the important role of pH in assessing local physiological states and monitoring metabolic abnormalities, we constructed a pH-sensing fiber by coating the CNT surface with a layer of polyaniline as the sensitive element, exploiting its stable redox transitions between emeraldine salt and base states at different pH values (Fig. 3c). Eventually, the resulting biosensing fibers all demonstrated comprehensive improvements in long-term stability under gradient concentrations for over 6 hours, which also validated the versatility of the optimization strategy (Fig. 3d–g). The satisfactory consistent and stable performance of the electrodes further confirms the repeatability and reliability of our optimizing strategy (Fig. S26).

The performances of the as-fabricated biosensing fibers were further systematically characterized *in vitro*. Figure 3h–k shows the current responses of fiber-shaped amperometric enzymatic sensors under physiologically relevant target analyte concentrations. The currents were highly linearly proportional to each biomarker, with sensitivities of 0.31 nA/µM for glucose sensors, 65.26 nA/mM for lactate sensors and 3.01 nA/µM for uric acid sensors. In addition, the sensitivity of the pH sensor exhibited a linear rise in open-circuit potential, with concentrations ranging from 7.0 to 4.0, with a sensitivity of 53.52 mV/pH. At the same time, each sensor demonstrated satisfactory selectivity, with negligible responses to other analytes at physiologically relevant concentrations in sweat (Fig. S27). As such, our fiber functionalization strategy incorporates inert analogous composite materials as a protective layer, effectively preserving critical sensing performances while demonstrating considerable reproducibility and enhanced long-term stability (Fig. 3l–o). Notably, these biosensing fibers also exhibited acceptable washability, maintaining stable electrochemical responses over 20 repeated washing cycles, further demonstrating the robustness of the functionalized layer on the fiber surface (Fig. S28).

### On-body sweat monitoring by the fabric system

**Figure 4.**
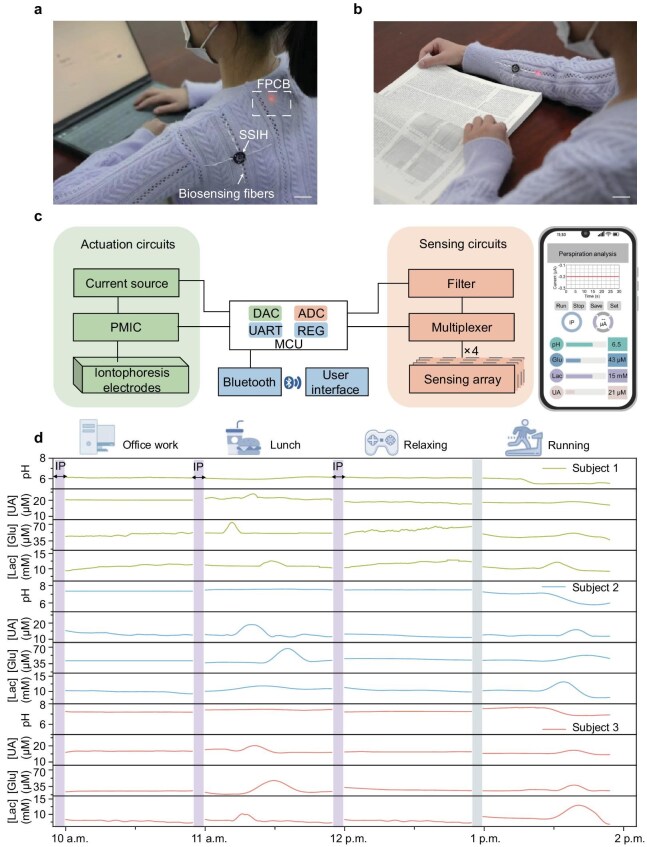
On-body demonstration and evaluation of the integrated electrochemical fabric biosensing system for continuous cross-activity sweat analysis. (a) Photograph of a subject wearing the electrochemical fabric on the back while performing office work (FPCB, flexible printed circuit board). Scale bar, 3 cm. (b) Photograph of a subject wearing the electrochemical fabric on the forearm while reading. Scale bar, 2 cm. (c) System-level block diagram and custom mobile application for autonomous iontophoresis and real-time data display (DAC, digital-to-analog converter; ADC, analog-to-digital converter; UART, universal asynchronous receiver-transmitter; REG, regulator; PMIC, power management integrated circuit). (d) Continuous cross-activity sweat monitoring in three healthy subjects under office work, lunch, relaxing and running conditions, with iontophoresis sweating performed at three intervals.

To validate the feasibility of portable health monitoring and interaction, we integrated the SSIH-based iontophoresis unit and biosensing fibers into various close-fitting areas of conventional garments (Fig. 4a and b, and Fig. S29). The system enables continuous physiological signal monitoring during sedentary activities, such as reading or desk work, with slight interference to the users' normal routines. A custom-developed flexible printed circuit board serves as the core component of the wearable biosensing system, enabling comprehensive system-level functionalities, including programmable iontophoretic actuation, *in situ* electrochemical signal processing and wireless interaction with the user interfaces via Bluetooth (Fig. 4c). As a proof of concept, long-term cross-activity monitoring of multiplexed biomarkers under sedentary scenarios was achieved by using our integrated wearable system (Fig. 4d). During three sedentary activities spanning office work, lunch breaks and gaming sessions, the glucose-concentration profile revealed characteristic metabolic responses, showing a marked elevation from baseline during the lunch period, followed by a gradual return to basal levels during the subsequent relaxation phase [61,62]. Lactate levels remained stable, indicating low physical exertion during these predominantly sedentary daily routines [63,64]. Uric acid levels are transiently elevated during the lunch break, aligning with the known impact of dietary protein intake on purine metabolism [65]. Additionally, we further validated the applicability for dynamic conditions and active users in a running scenario, observing no significant fluctuations in data transmission (Fig. S30). Moreover, the accuracy of our multiplexed sweat-sensing measurements was further validated through parallel analysis by using liquid chromatography-tandem mass spectrometry (LC-MS/MS) (Fig. S31). In general, our wearable electrochemical fabric biosensing system demonstrated remarkable reliability and potential in personalized health monitoring and metabolic disorder management.

## CONCLUSION

Here, we have developed a comfortable iontophoretic electrochemical fabric biosensing system with a long-term continuous stability of over 6 hours. The iontophoresis based on the SSIH unit enables effective sweat induction through ultra-low-current stimulation by optimizing the interface impedance. Additionally, incorporating a stabilized transduction layer design on the biosensing fibers mitigates the degradation under prolonged operation, significantly enhancing long-term stability for reliable monitoring over a 6-hour period. The strategy allows autonomous perspiration sampling, dynamic sensing analysis and wireless data communication, enabling reliable health monitoring. However, challenges still exist, including adaptability to skin variations, sweat refreshment and sample fouling, and linking *in situ* data to health conditions. Future optimizations could involve integrating high-precision skin impedance sensors for *in situ* testing combined with the programmable iontophoresis modules to enhance user acceptance. Moreover, such long-term monitoring also presents opportunities in cloud storage and AI for diving into sweat and advancing personalized healthcare.

## METHODS

The detailed materials and methods are available in the online Supplementary data.

## Supplementary Material

nwaf155_Supplemental_File
