# Supplementary material for: An autonomous fabric electrochemical biosensor for efficient health monitoring
Source: Natl Sci Rev. 2025 Apr 23;12(6):nwaf155. doi: 10.1093/nsr/nwaf155 (PMC12118457; doi:10.1093/nsr/nwaf155)
Supplement: nwaf155_Supplemental_File [file nwaf155_supplemental_file.pdf]

Supplementary Information

An Autonomous Fabric Electrochemical Biosensor for Efficient Health Monitoring

Liangliang Zhou[1,2†], Changxin Li[2,†], Yongfeng Luo[1,*], Qimin Liang[2], Yangyang Chen[2], Zhuojun Yan[2], Longbin Qiu[3,*], Sisi He[2,*]

[1]Hunan Province Key Laboratory of Materials Surface & Interface Science and Technology, College of Electronic Information and Physics, Central South University of Forestry and Technology, Changsha 410004, China;

[2]Shenzhen Key Laboratory of Flexible Printed Electronics Technology, School of Science, Harbin Institute of Technology (Shenzhen), University Town, Shenzhen 518055 China;

[3]Department of Mechanical and Energy Engineering, SUSTech Energy Institute for Carbon Neutrality, Southern University of Science and Technology, Shenzhen 518055, China

***Correspondence authors.** Emails: hesisi@hit.edu.cn; qiulb@sustech.edu.cn; yfluo@csuft.edu.cn

[†]Equally contributed to this work.

Experimental Section

Materials

Aniline (C_6H_7N, 99%), sodium phosphate dibasic dodecahydrate ($Na_2HPO_4 \cdot 12H_2O$, 99%), sodium dihydrogen phosphate monohydrate ($NaH_2PO_4 \cdot H_2O$, 98%), polyvinyl alcohol (PVA, 99%), N, N'-methylene bisacrylamide (MBAA, 99%), 2, 2′-azobis(2-methylpropionamidine) (AIBA, 97%), acrylic acid (AA, 99%), acetic acid (CH_3COOH, 99.7%), chitosan ($C_6H_{11}NO_4X_2$, 95%), single-walled carbon nanotube (SWCNT, 90%), glucose oxidase (50 U/mg), potassium ferricyanide ($K_3[Fe(CN)_6]$, 99%), potassium chloride (KCl, 99%), sodium chloride (NaCl, 99.99%), magnesium chloride ($MgCl_2$, 99%), ammonium chloride (NH_4Cl, 99%), poly(3,4-ethylenedioxythiophene)-poly(styrenesulfonate) (PEDOT:PSS, 99%), D-glucose ($C_6H_{12}O_6$, 99%), polyvinyl butyral (PVB, 99%), calcium chloride ($CaCl_2$, 97%), N,N-Dimethylformamide (DMF, 25%), and iron chloride hexahydrate ($FeCl_3 \cdot 6H_2O$, 99%) were purchased from Aladdin (Shanghai, China). Phosphate-buffered saline solution (pH = 7.4) was purchased from Sangong Bioengineering Co., Ltd. Lactate Oxidase (250 U/mg) was purchased from Shanghai Yuanye Biological Co., Ltd. Sulfuric acid (H_2SO_4, 25%) and hydrochloric acid (HCl, 36%) were obtained from Guangzhou Chemical Reagent Factory. L-lactate ($C_3H_6O_3$, 85%) was purchased from Tianjin Xiensi Biochemical Technology Co., Ltd. Silicone fiber was purchased from Smooth-On Inc. Uricase (20 U/mg) and Nickel (II) Chloride ($NiCl_2$, 42%) were purchased from Tansoole. Urea (CON_2H_4, 98%) was purchased from General-reagent, and uric acid (UA, 98%), polyacrylonitrile (PAN, 99%) and silicon dioxide (SiO_2, 2.5%) were purchased from Adamas-Beta. The deionized water used was purified from MilliQ device from Millipore. Unless specifically mentioned, all the chemical reagents were used directly without further purification.

Preparation of SSIH

In a typical synthesis, 20% w/w PVA and 10% w/w carbachol were mixed and heated at 100°C in an oil bath under stirring for 1 h until completely dissolved. The homogeneous solution was then poured into a silicone mold, frozen at -20°C for 8 h, and subsequently thawed at room temperature for 3 h to form the PVA hydrogel. Next, the pre-formed PVA hydrogel was first immersed in an aqueous solution containing AA monomers, MBAA as the crosslinker, and AIBA as the initiator for 10 minutes. Then, it was heated in an oil bath at 70°C under magnetic stirring at 400 rpm for 1 hour, allowing the polymerization of AA within the PVA matrix. Subsequently, the precursor was poured into a mold, frozen at -20°C for 24 h, and then thawed again at room temperature for 3 h to obtain anode hydrogel. The hydrogel without the addition of polyacrylic acid (PAA) was used as control samples. The preparation of the cathode hydrogel follows the same procedure, except that carbachol is replaced with NaCl,

to ensure conductivity and facilitate the iontophoretic process. The ring-shaped SSIH electrodes were assembled using the as-prepared hydrogels, with the anode diameter of 1 cm and the cathode diameter of 0.5 cm using CNT sheets as electrodes on top of them.

Electrochemical impedance characterization

A two-electrode system was employed for skin impedance testing using an electrochemical workstation (CHI660E). Two identically sized SSIH electrodes (2 cm in diameter, 1 mm in thickness) served as the working and counter electrodes. The hydrogel electrodes were placed on the skin, and impedance measurements were conducted across a frequency range of 10^{-1} to 10^{6} Hz.

Mechanical characterization

Tensile tests were performed using a universal testing machine (HY-0580, Shanghai Hengyi Test Instrument Co., Ltd.). The hydrogels were cut into dumbbell-shaped specimens with dimensions of 100 mm in total length, 5 mm in narrow width, 20 mm in narrow length, and 25 mm in broad width. The tensile tests were conducted at a constant speed of 100 mm/min. Adhesion tests were conducted using flat, smooth hydrogel films with dimensions of 2.5 cm × 1 cm × 1 mm. An acrylate adhesive was applied to attach a polyethylene terephthalate film to the back of the porcine skin. The film was applied to prevent any deformation caused by stretching during the test. The hydrogel films were then adhered between the porcine skins. The adhesion strength was evaluated using the standard lap shear test with a constant peeling speed of 50 mm/min. Each sample was tested in triplicate to ensure consistency and reliability.

Fabrication and preparation of the electrochemical fabric biosensors

Preparation of CNT fiber: Firstly, aligned CNT fibers with a width of 500 μm were prepared using the floating chemical vapor deposition method[1]. Then, three CNT fibers were aligned in parallel and twisted together using a motor-driven system at a rotation speed of 1500 rpm to form the secondary helical structure with multi-scaled aligned channels as electrode substrate. Before use, the helical CNT fibers were activated in 0.1 M H_2SO_4 solution through cyclic voltammetry between -1.5 V and 1.5 V at 0.05 V/s scan rate for 10 cycles. The activated fibers were rinsed three times with deionized water and dried at room temperature.

Fabrication of working fiber electrodes*:* The enzyme-based fiber electrodes were constructed with a layered structure comprising the CNT fiber, PB transducer layer, NiHCF protective layer, and enzyme biorecognition element. The PB transducer layer was electrodeposited onto the CNT electrodes by cyclic voltammetry in a solution consisting of 2.5 mM $FeCl_3$, 2.5 mM $K_3Fe(CN)_6$, 100 mM KCl, and 100 mM HCl. The deposition was performed

by applying an optimized number of cycles for each electrode type, with a scan rate of 100 mV/s and a potential range of -0.2 to 1 V versus Ag/AgCl. Subsequently, a NiHCF protection layer was then electrodeposited onto the electrodes using cyclic voltammetry in a solution containing 0.5 mM $NiCl_2$, 0.5 mM $K_3Fe(CN)_6$, 100 mM KCl, and 100 mM HCl. The deposition was carried out by applying cyclic voltammetry with a potential range of 0 to 0.8 V versus Ag/AgCl and a scan rate of 100 mV/s for an optimized number of cycles. After drying, 3 μL of chitosan/SWCNT/enzyme mixture was drop-coated onto the PB/CNT electrode. Finally, a 0.5 wt% Nafion membrane was coated as an outer layer for the enzyme-based electrodes to minimize interference, and stored overnight before use. The chitosan/SWCNT/enzyme mixture was prepared by dissolving chitosan in 1% (w/w) acetic acid under magnetic stirring, followed by SWCNT dispersion through stirring at 40°C for 1 hour and ultrasonication. Subsequently, glucose oxidase, lactate oxidase, or uric acid oxidase was individually mixed with the chitosan/SWCNT solution to achieve an enzyme concentration of 40 mg/mL. As for the preparation of the pH-sensing electrode, polyaniline was electrodeposited onto the CNT fiber in a solution containing 0.1 mol/L aniline and 0.1 mol/L H_2SO_4 using cyclic voltammetry from 0.2 to 1.0 V for 25 cycles at a scan rate of 100 mV/s.

Assembly of biosensing fibers: Firstly, silicone fibers were fabricated by injecting the Ecoflex mixture into a polyethylene terephthalate tube, followed by peeling it off and curing at room temperature for 4 hours. Subsequently, each biosensing fiber was formed by coaxially twisting a functionalized CNT-based working electrode and a Ag/AgCl reference/counter electrode within a stretchable silicone fiber, ensuring adaptability to an extensive stretchability of up to 300% for more than 2000 cycles (**Figure S1**). The Ag/AgCl electrode was prepared by applying commercial Ag/AgCl ink onto a CNT fiber through dip-coating method with the electrical conductivity of 1.0 x 10^5 S/m. Moreover, each biosensing fiber was coupled with a super-hydrophilic PAN/SiO_2 composite layer on the outer periphery, serving as a sweat-absorbing layer. The fabrication procedure of sweat-absorbing layer via electrospinning was as follows: 1 g PAN and 0.1 g SiO_2 were dissolved in 11.1 ml DMF and stirred at 50°C for 4 h to form a 9% mixed solution. The PAN/SiO_2 spinning solution was transferred into a syringe and electrospun under controlled conditions. The syringe needle tip was positioned 15 cm from the collector, while a positive voltage of 20 kV was applied. The solution was fed at a rate of 0.5 ml/h, and the collector roller maintained a rotation speed of 500 rad/min. After 4h of spinning, the PAN/SiO_2 sweat-absorbing layer was obtained.

In vitro sensor characterization*:* The electrochemical characterization of the biosensing fibers was evaluated in a 0.01 M PBS (pH 7.0) with change concentration of analyte, with glucose ranging from 0 to 100 μM, lactate ranging from 0 to 15 mM, UA ranging from 0 to 100 μM, and pH ranging from 4 to 7. All the in vitro characterizations were conducted using

amperometric and potentiometric techniques via a multichannel electrochemical workstation (CHI660E, CH Instruments Inc.). To investigate the reproducibility of the sensors, the electrochemical responses of multiple samples fabricated in different batches were compared. To characterize the stability of the sensors, a 6-hour test was conducted by starting at zero concentration, progressively increasing the concentration, and then reducing it back to near-zero levels. The selectivity of the biosensing fibers was evaluated by introducing physiologically relevant concentrations of common sweat constituents into a standard solution and recording the electrochemical response. The washing stability of the electrochemical fabric was tested through a 10-min machine washing process following international standards (ISO 105/C10:2006 and AATCC 612013). The stability of PB/CNT and NiHCF/PB/CNT electrodes was evaluated in 0.01 M PBS (pH 7.0) at different pH values by performing cyclic voltammetry scans from -0.2 V to 1.0 V at a scan rate of 50 mV/s. Moreover, The potential stability of the Ag/AgCl electrodes were assessed through open circuit potential measurements in KCl solutions of different concentrations (10 mM, 100 mM, and 1 M), compared with that of a commercial Ag/AgCl electrode (Tianjin Aida Hengsheng Technology Co., Ltd).

Characterization: All electrochemical characterization was performed with a CHI660E workstation (CHI660E). The morphology of sweat-absorbing layer and hydrogels was characterized by scanning electron microscope (SEM, Zeiss Crossbeam 350). The hydrophilic properties of the sweat-absorbing layer were evaluated using a contact angle meter (SDC-220SH), with a water droplet volume of 3 μL for the test. Fourier transform infrared spectroscopy was conducted on a Thermo Nicolet 6700 spectrometer in the range of 4000-1500/cm^{-1} using KBr pellets. Fluorescence microscopy (Leica, TCS SP8) was used to evaluate ex vivo transdermal delivery on porcine skin, and Rhodamine B was loaded into the anode as a model drug for visualization. The electrolyte concentrations in sweat samples were analyzed using a liquid chromatography-tandem mass spectrometry (LC-MS/MS, Agilent 1290II-6460). The pH values of sweat samples were measured using a pH meter (Leici PHS-3E). Digital photographs were taken using a Sony ILCE-A7M4 camera.

On-body evaluation of electrochemical fabric biosensing system

Subject recruitment: The study protocol was approved by the Medical Ethics Committee of Harbin Institute of Technology (HIT-2025011). All participants, three males and two females, were healthy volunteers without mental health conditions who provided written informed consent, with participation being entirely voluntary without compensation. Summary of healthy human subjects demographics is provided in **Table 2**.

Evaluation of Sweat induction unit: Before the experiment, the subject's skin was cleaned using alcohol wipes. The SSIH electrode being tested was placed on the subjects' forearms, and iontophoretic-induced sweating was achieved by applying a specified current through a source measure unit (Keithley, 2470) for 10 minutes with a 10 V upper voltage limit for safety protection against skin discomfort or burns. Each experiment was repeated three times, with at least one day between each experiment to ensure the reliability of the results. Photographs were recorded, and a water-sensitive dye was applied for visualization.

Evaluation of biosensing system during daily activities: To demonstrate the advantages of the electrochemical fabric biosensing system for on-demand continuous monitoring, biomarker monitoring across activities was conducted from 10:00 a.m. to 2:00 p.m. The protocol involved administering three sweat induction sessions through the skin-interfaced unit before transitioning between different sedentary activities, including office work, lunch, and gaming relaxation. In the final hour, participants were instructed to perform running to evaluate the system's performance in active users or exercise scenarios. Data were wirelessly transmitted via Bluetooth indications, while the ambient temperature was maintained at 25°C throughout the test. Additionally, sweat samples were collected and stored at -20°C for subsequent validation using both electrochemical biosensors and commercial analytical instruments.

Supplementary Figures and Tables

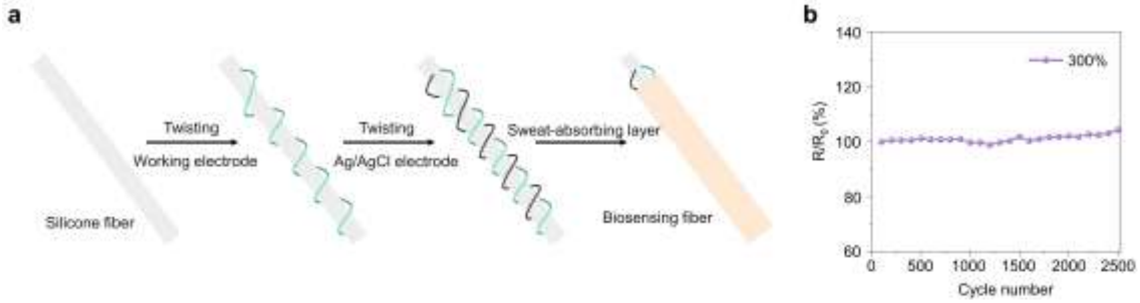

Figure S1. (a) Illustration of the biosensing fiber assembly processes. (b) Relative resistance change curves of biosensing fiber under 300% tensile deformation.

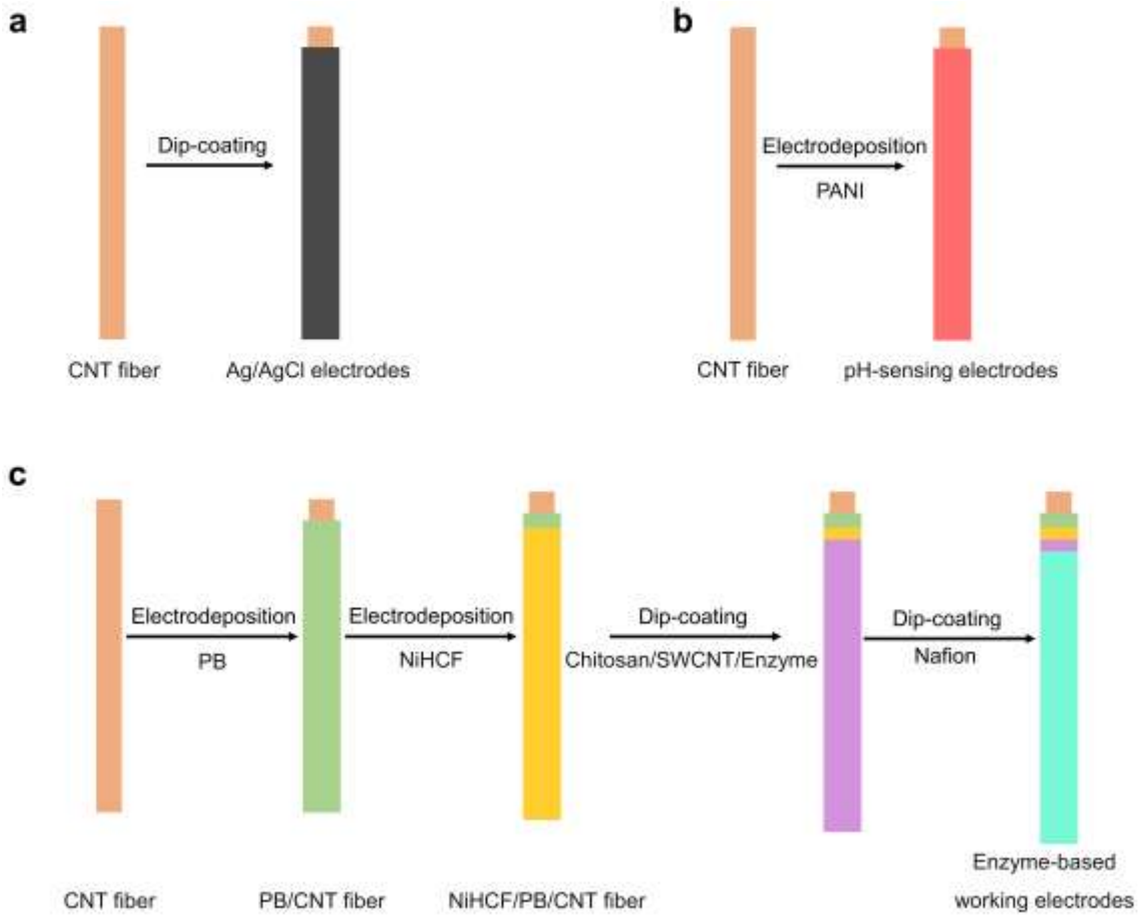

Figure S2. Illustration of the fabrication processes of (a)Ag/AgCl electrodes, (b) pH-sensing electrodes, (c) enzyme-based working electrodes.

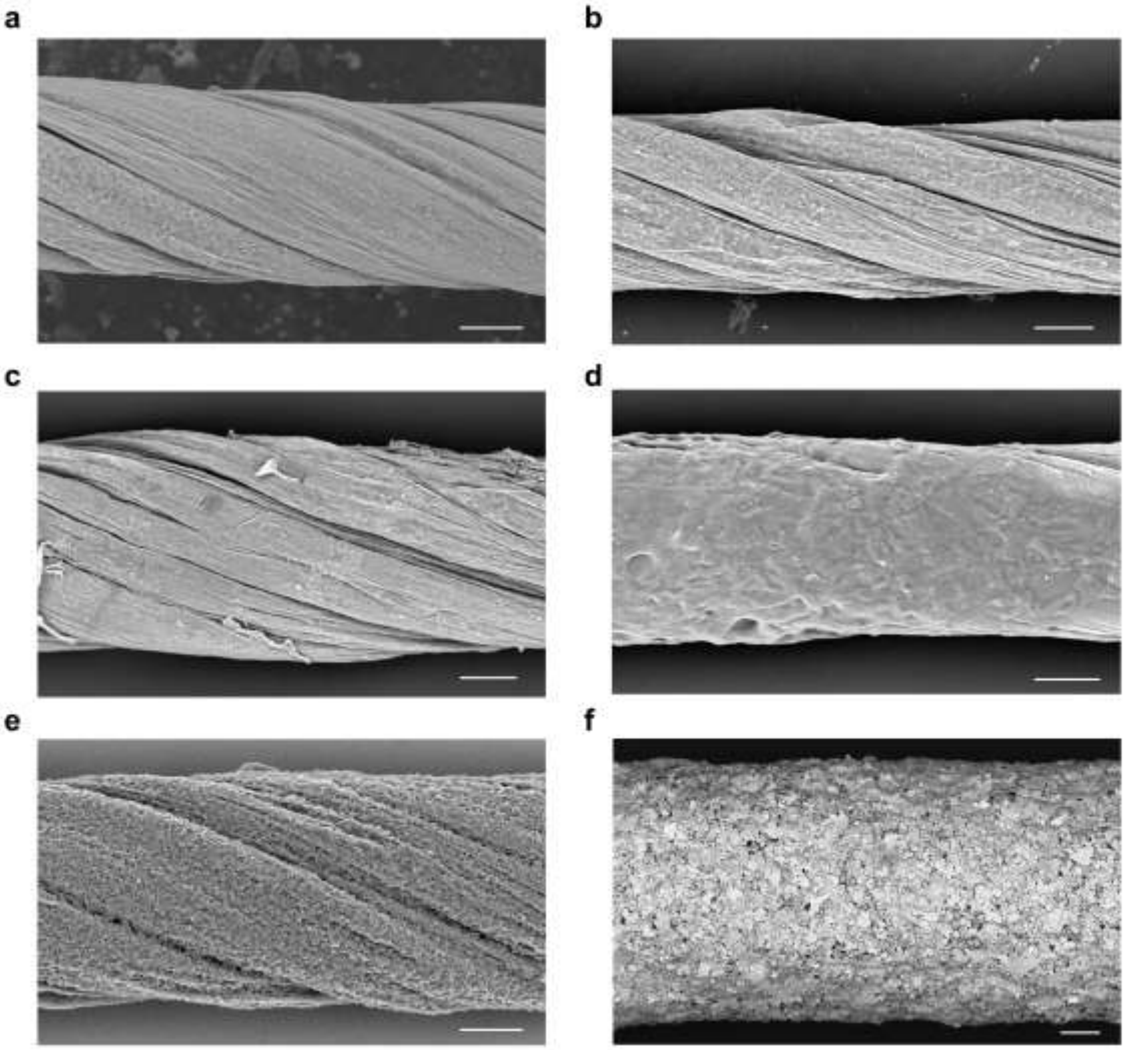

Figure S3. SEM images of the initial CNT electrode (a), PB/CNT electrode (b), NiHCF/PB/CNT electrode (c), enzyme-based working electrodes (d), pH-sensing electrode (e), and Ag/AgCl electrode (f). Scale bar, 25 µm.

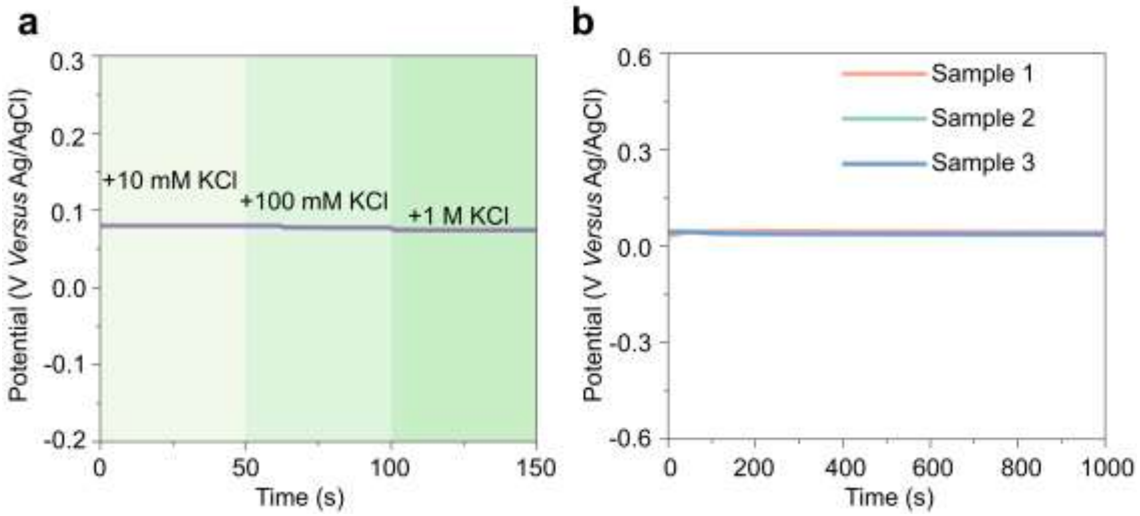

Figure S4. (a) The open circuit potential-time curves of Ag/AgCl electrodes. (b) Three reproductions of Ag/AgCl electrodes.

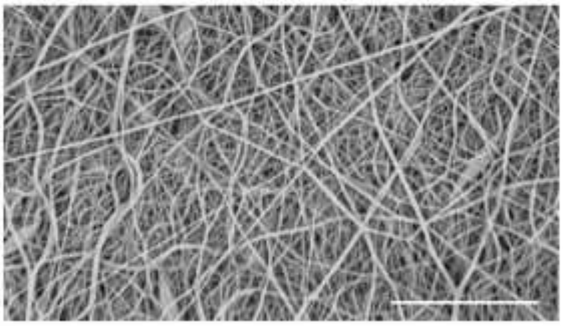

Figure S5. Scanning electron microscope image of PAN/SiO_2 sweat-absorbing layer. Scale bar, 15 µm.

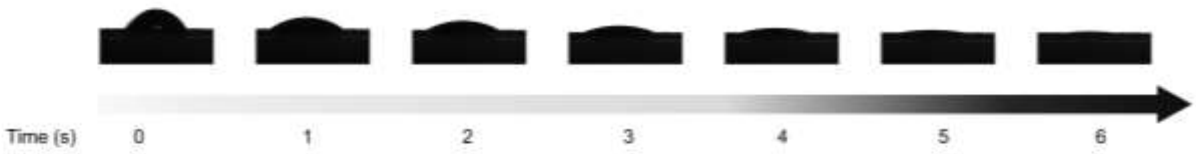

Figure S6. The contact angle change of the PBS droplet (3 μL) on PAN/SiO_2 sweat-absorbing layer.

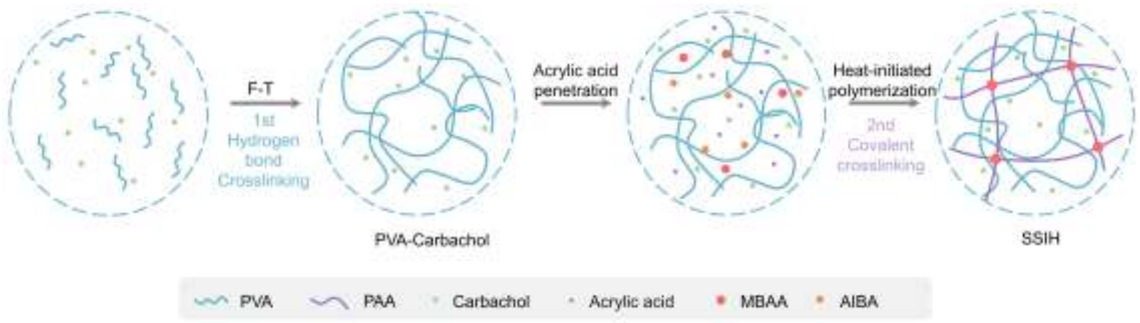

Figure S7. Schematic illustration of the synthesis procedures of SSIH.

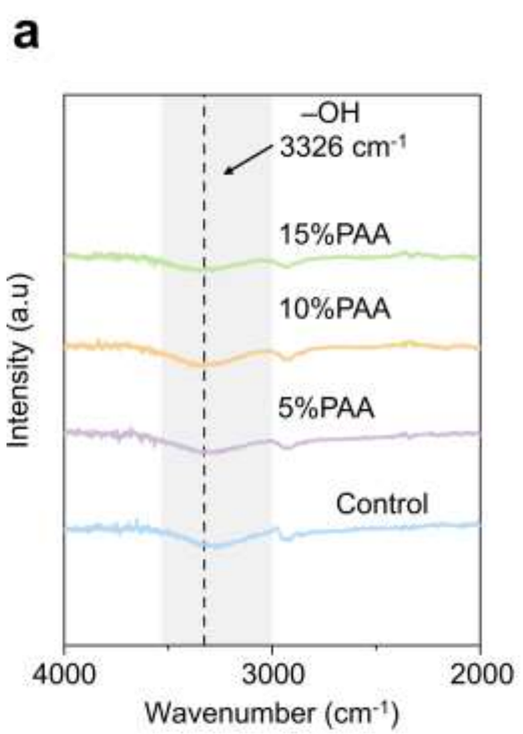

Figure S8. Fourier transform infrared spectroscopy spectra of hydrogels with different amounts of PAA.

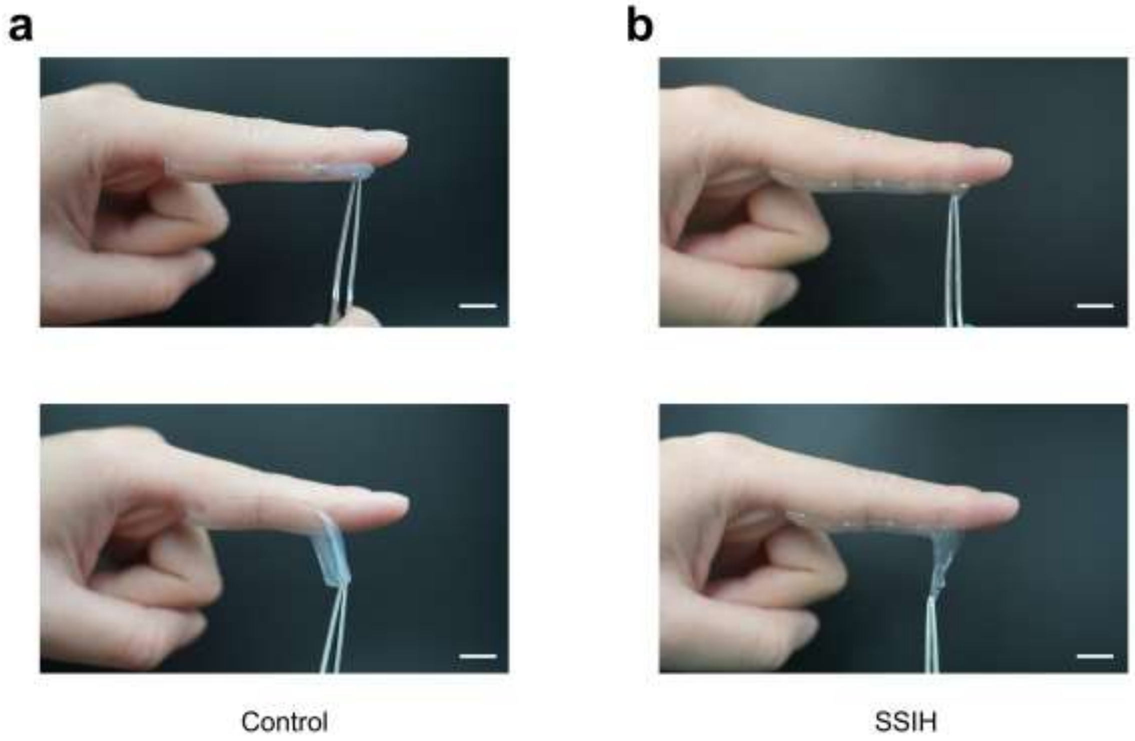

Figure S9. Photographs of peeling test of control (a) and SSIH (b) on finger skin. Scale bar, 1 cm.

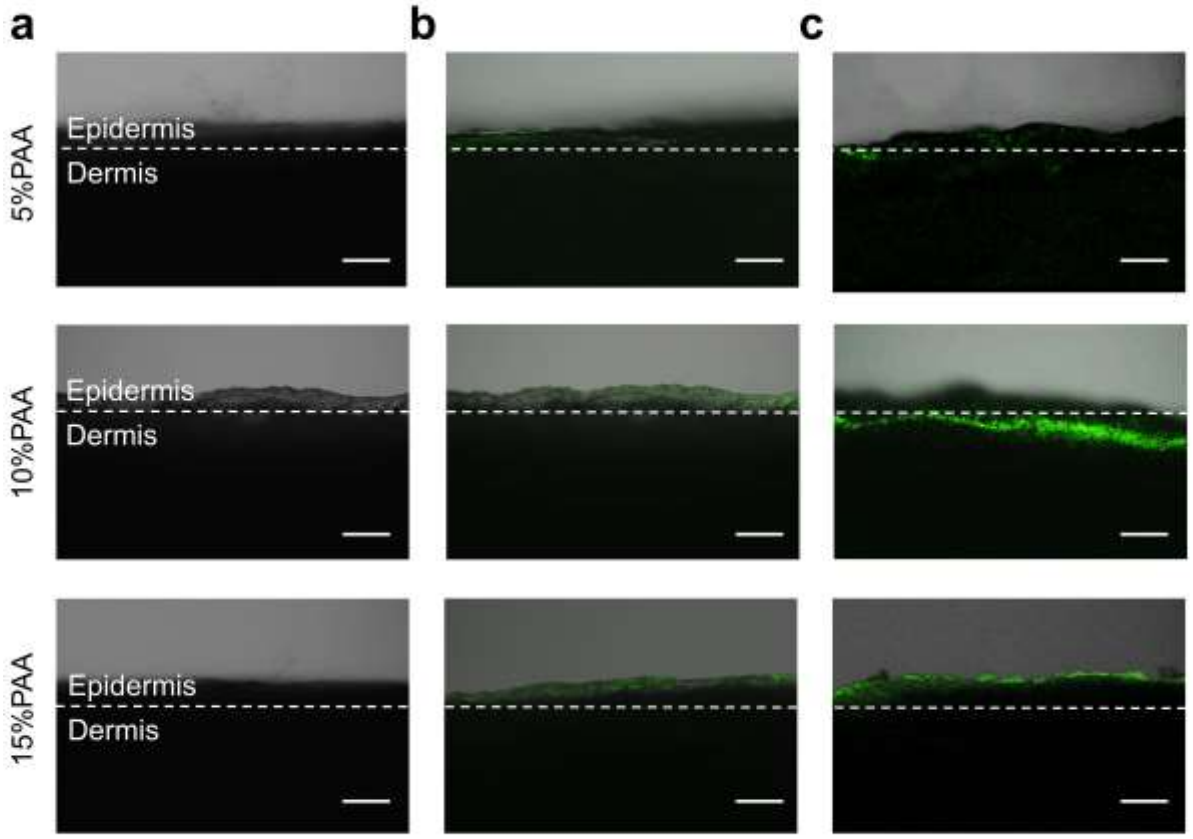

Figure S10. Fluorescence microscopy characterization of the iontophoretic transdermal drug delivery process using different hydrogels with increasing PAA content on porcine skin. (a) Control sample without drug applied. (b) After Rhodamine B loaded hydrogel was placed on porcine skin for 10 minutes. (c) After Rhodamine B loaded hydrogel was placed on porcine skin with iontophoresis process for 10 minutes. Scale bar, 250 µm.

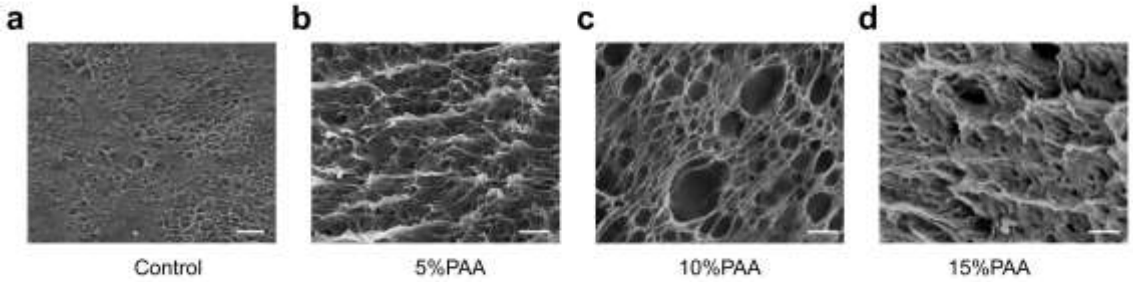

Figure S11. SEM images of (a) PVA, (b) PVA-5%PAA, (c) PVA-10%PAA, and (d) PVA-15%PAA hydrogel. The microstructure transitions from a dense, compact network in pure PVA hydrogel to an increasingly porous and interconnected structure as more PAA is incorporated. Scale bar, 1 µm.

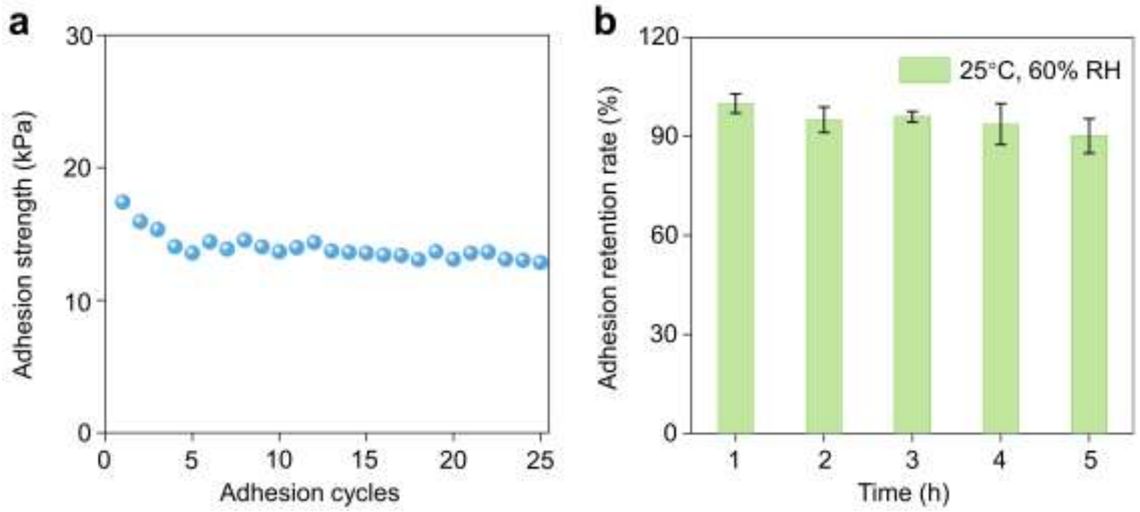

Figure S12. Long-term adhesion performance of SSIH under 25°C and 60% relative humidity (RH). (a) Adhesion strength over repeated lap shear tests. (b) Evolution of adhesion retention rate over time (n=3, mean ± s.d).

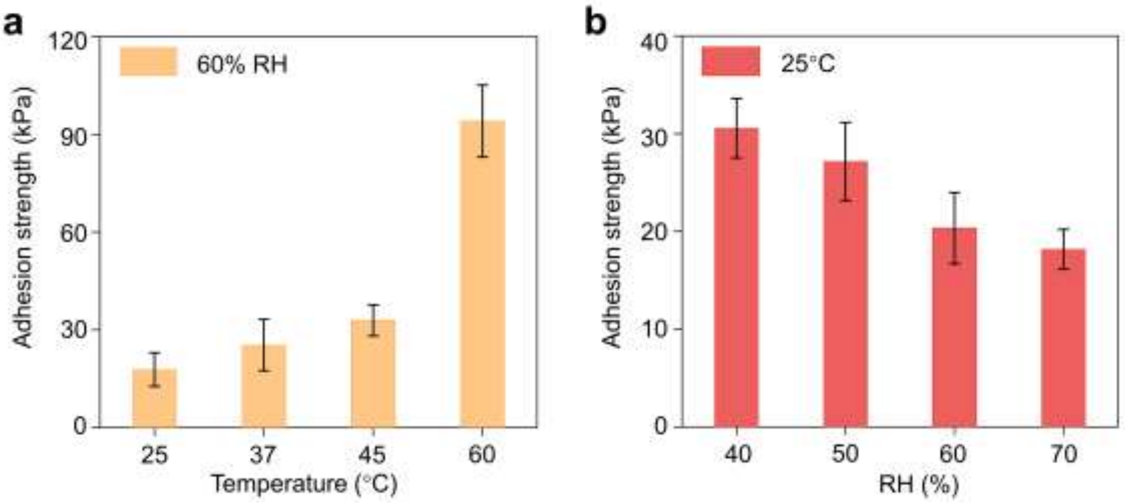

Figure S13. Adhesion strength of SSIH under varying environmental conditions. (a) At different temperatures under 60% RH (n=3, mean ± s.d). (b) At different RH levels under 25°C (n=3, mean ± s.d).

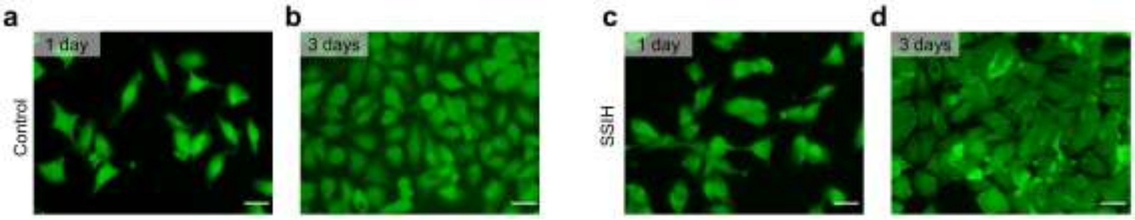

Figure S14. Fluorescence microscope images of hepatocytes after co-cultured with the control (a and b) and SSIH samples (c and d) for 1 day and 3 days respectively. Scale bar, 800 μm.

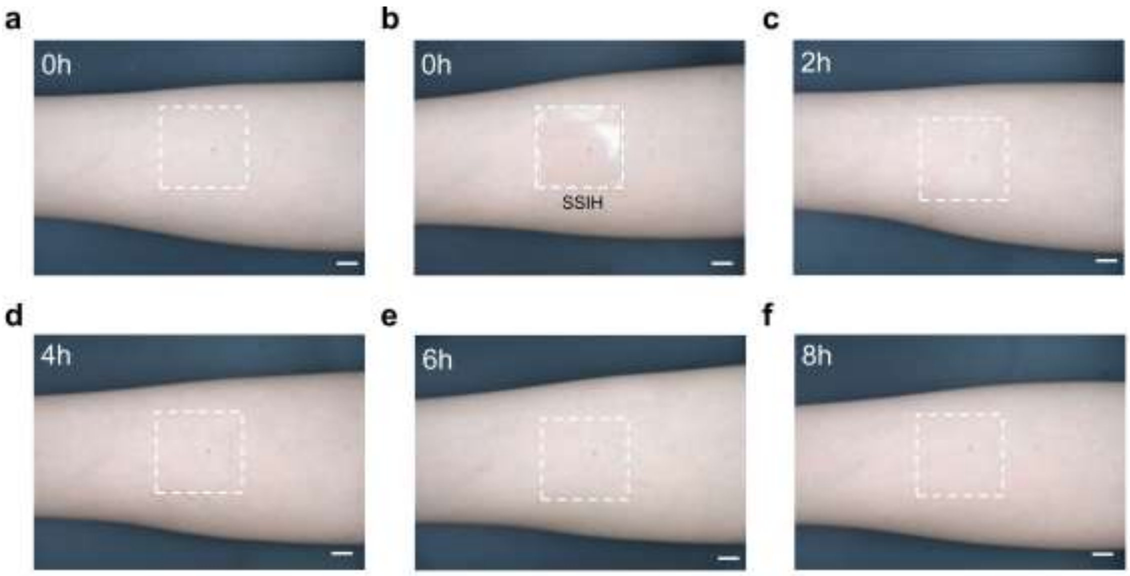

Figure S15. The optical image of skin condition during prolonged SSIH wear. Scale bar, 1 cm.

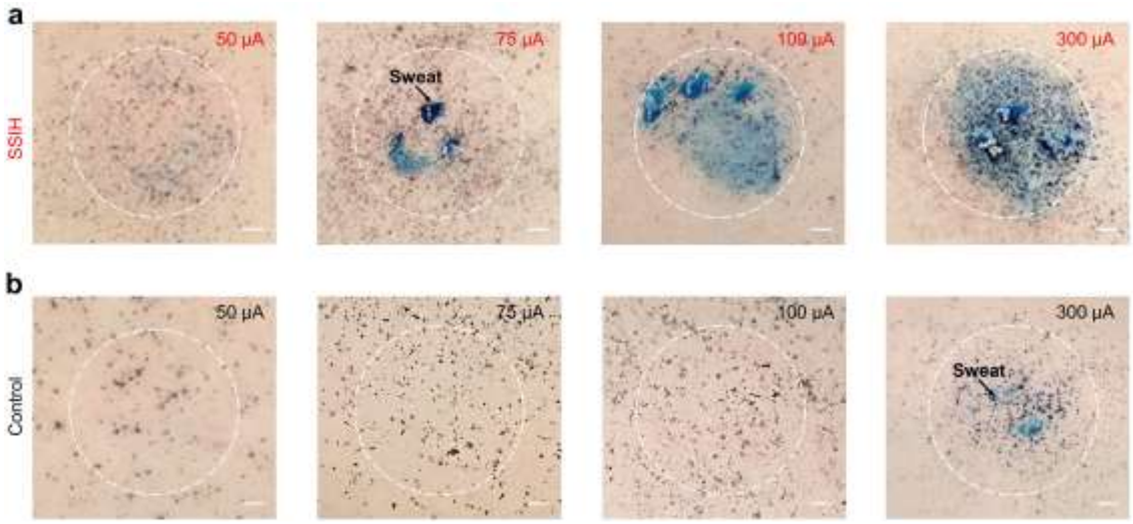

Figure S16. Comparison of sweat induction by iontophoresis based on control and SSIH under varying operational currents from 50 µA to 300 µA, with sweat secretion highlighted by blue dye staining. Scale bar, 2.5 mm.

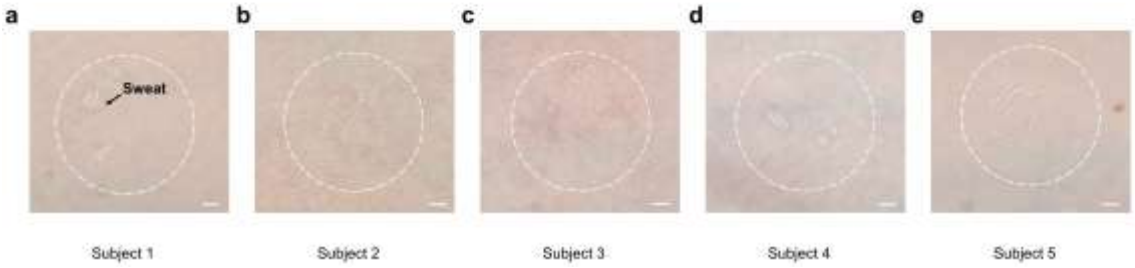

Figure S17. Optical images of iontophoresis-sweating in five subjects by SSIH electrodes. Scale bar, 2.5 mm.

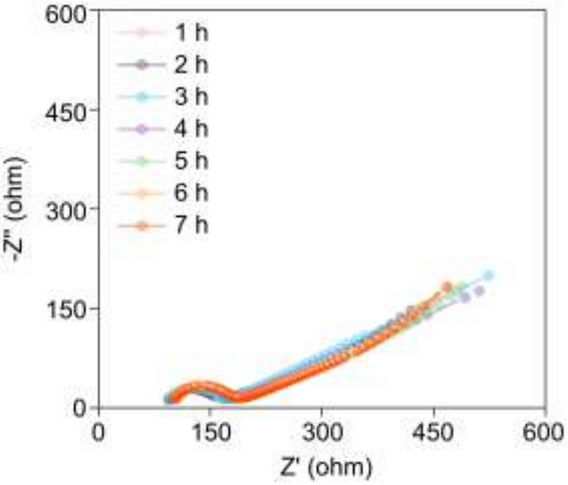

Figure S18. Evolution of interfacial impedance over time.

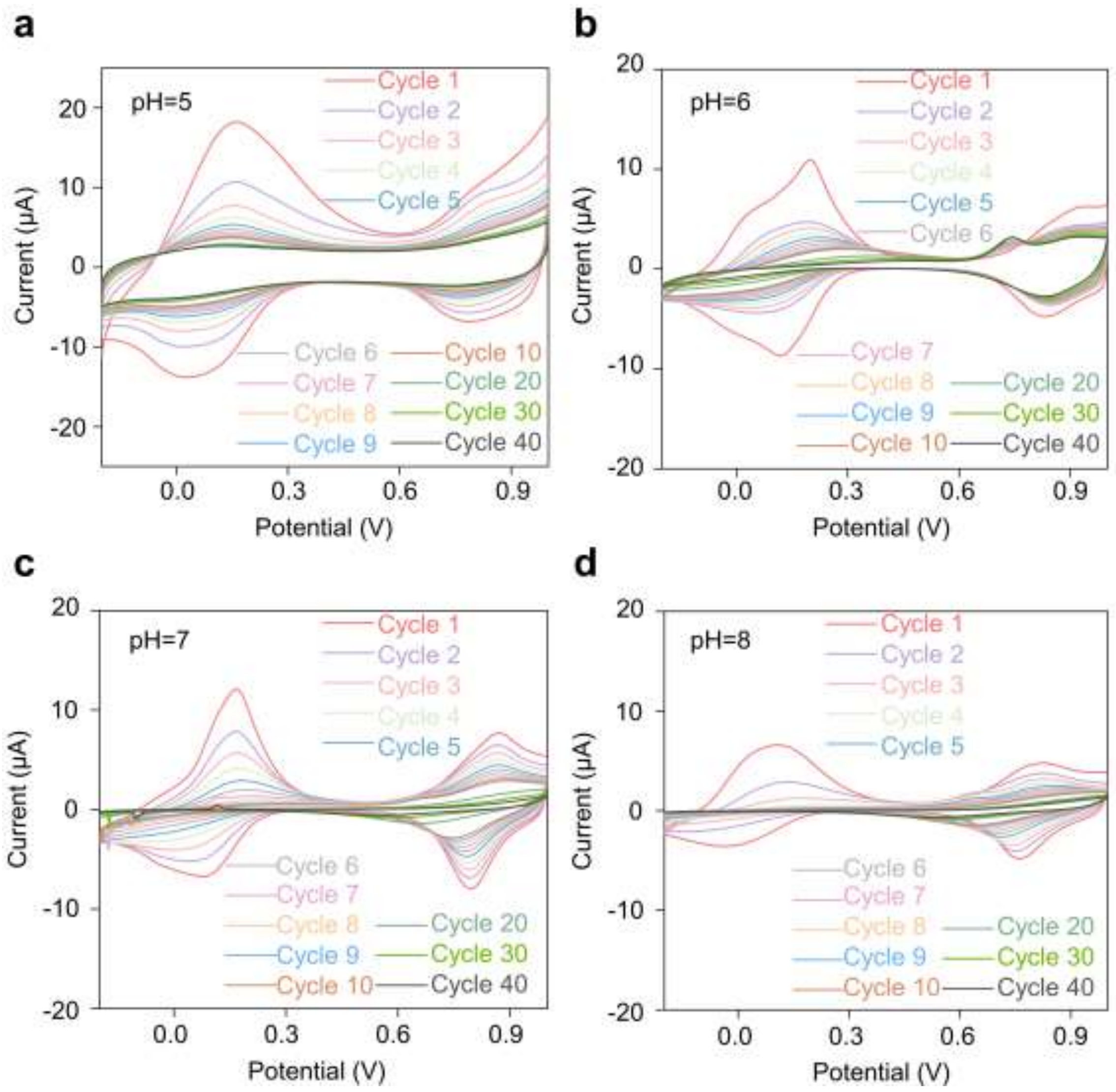

Figure S19. Cyclic voltammetry scans of PB/CNT electrodes at different pH values, scanned from -0.2 V to 1.0 V at a scan rate of 50 mV/s.

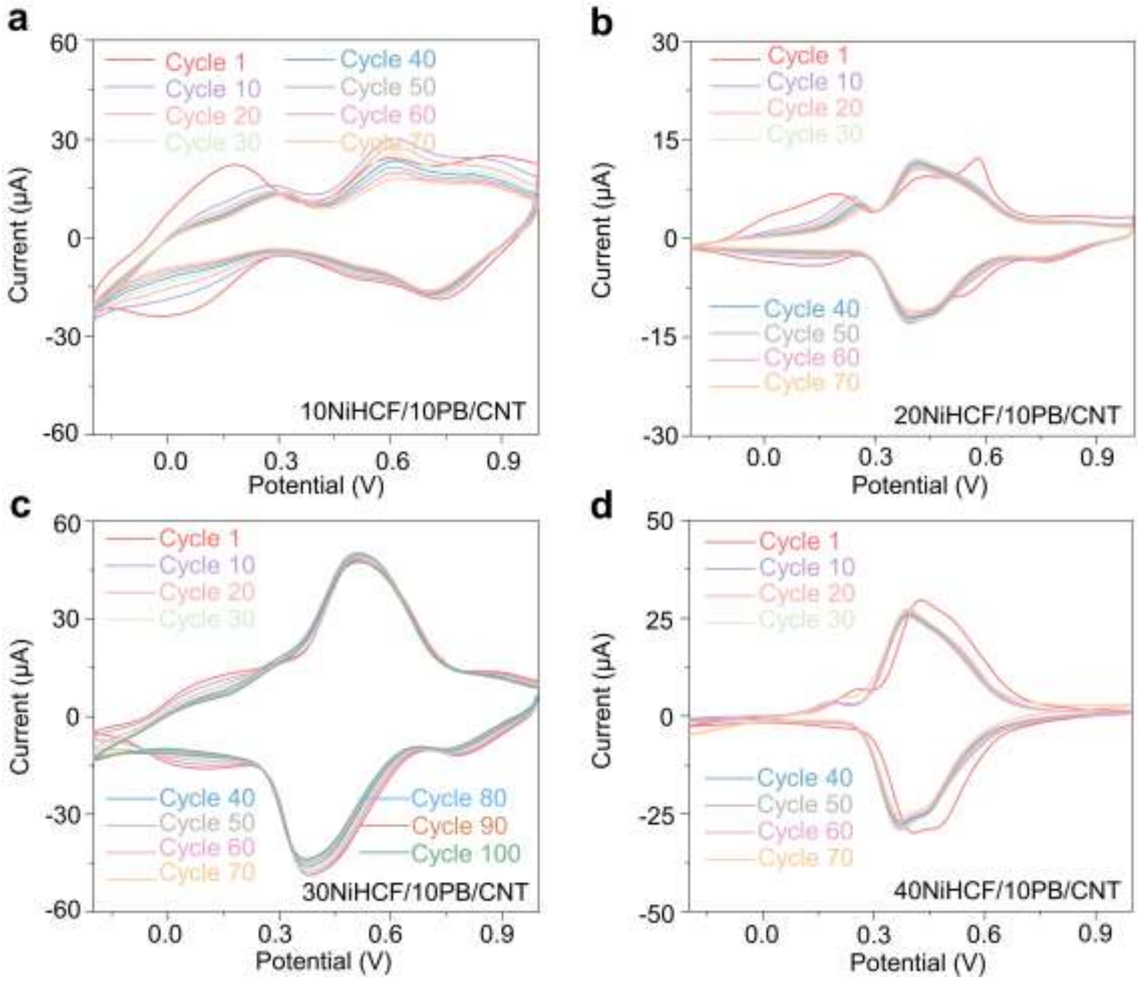

Figure S20. Cyclic voltammetry scans of PB/CNT electrodes deposition at different numbers of NiHCF layers.

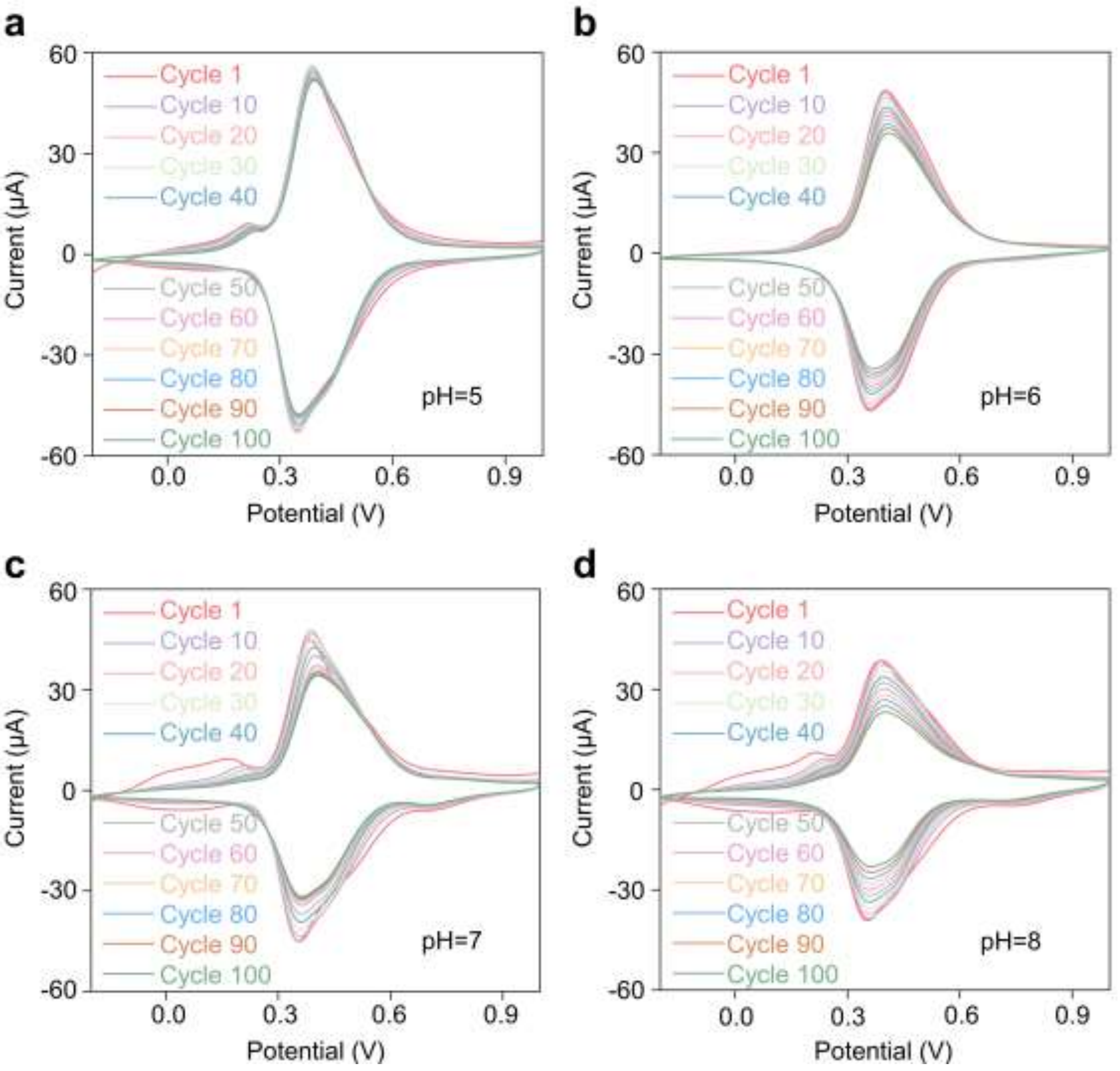

Figure S21. Cyclic voltammetry scans of NiHCF/PB/CNT (30 cycles NiHCF deposited on 10 cycles PB) electrodes at different pH values, scanned from -0.2 V to 1.0 V at a scan rate of 50 mV/s.

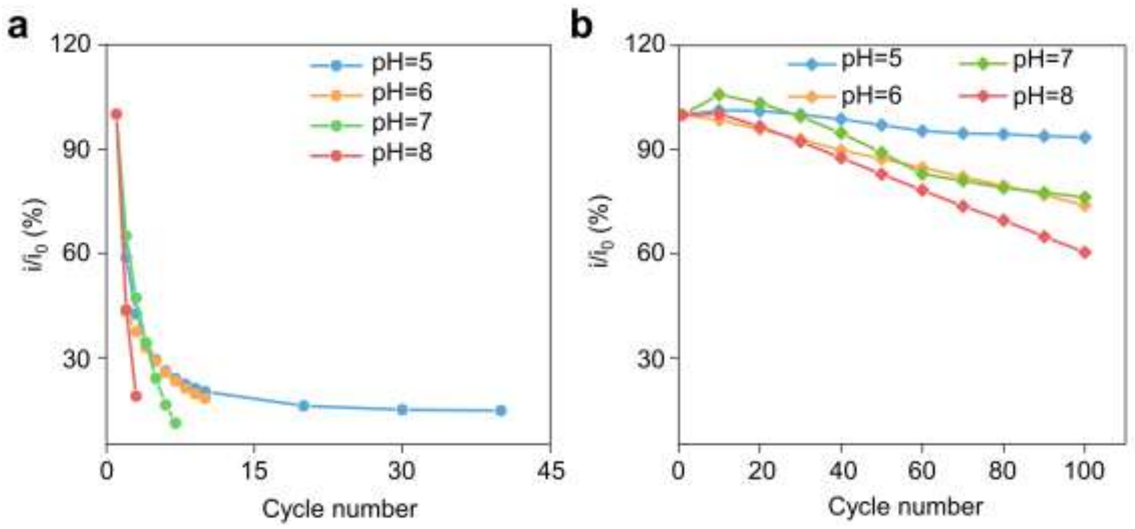

Figure S22. Changes in the oxidation peak current of PB/CNT electrodes (a) and NiHCF/PB/CNT electrodes (b) under repetitive CV scans. i and i_0 represent the peak current height and the initial first scan value, respectively.

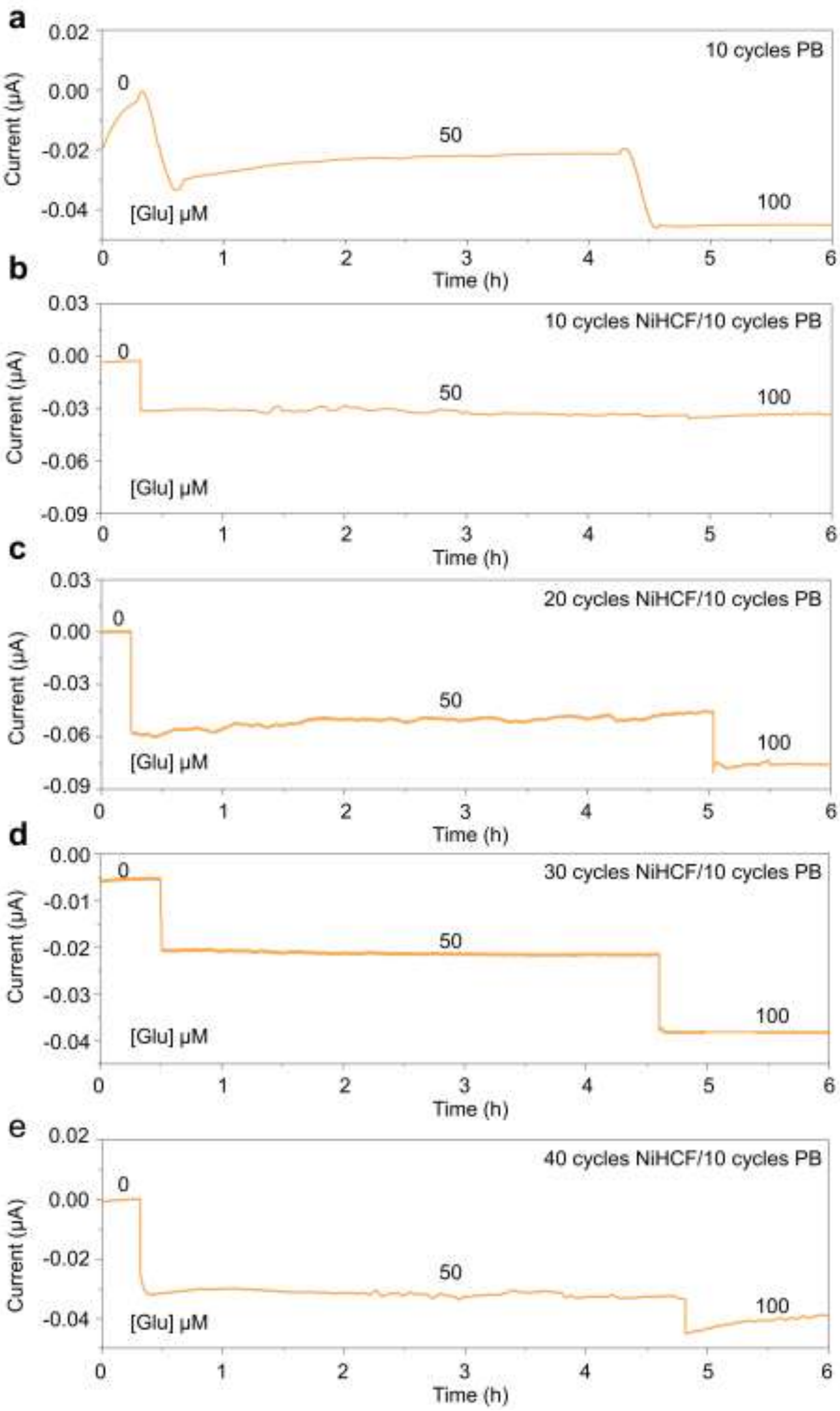

Figure S23. The long-term stability test for glucose biosensing fibers prepared with varying NiHCF deposition cycles. Amperometric responses of glucose biosensing fibers based on PB (a), PB with 10 cycles of NiHCF (b), PB with 20 cycles of NiHCF (c), PB with 30 cycles of NiHCF (d), and PB with 40 cycles of NiHCF (e) in PBS solutions containing 0, 50, 100 μM glucose.

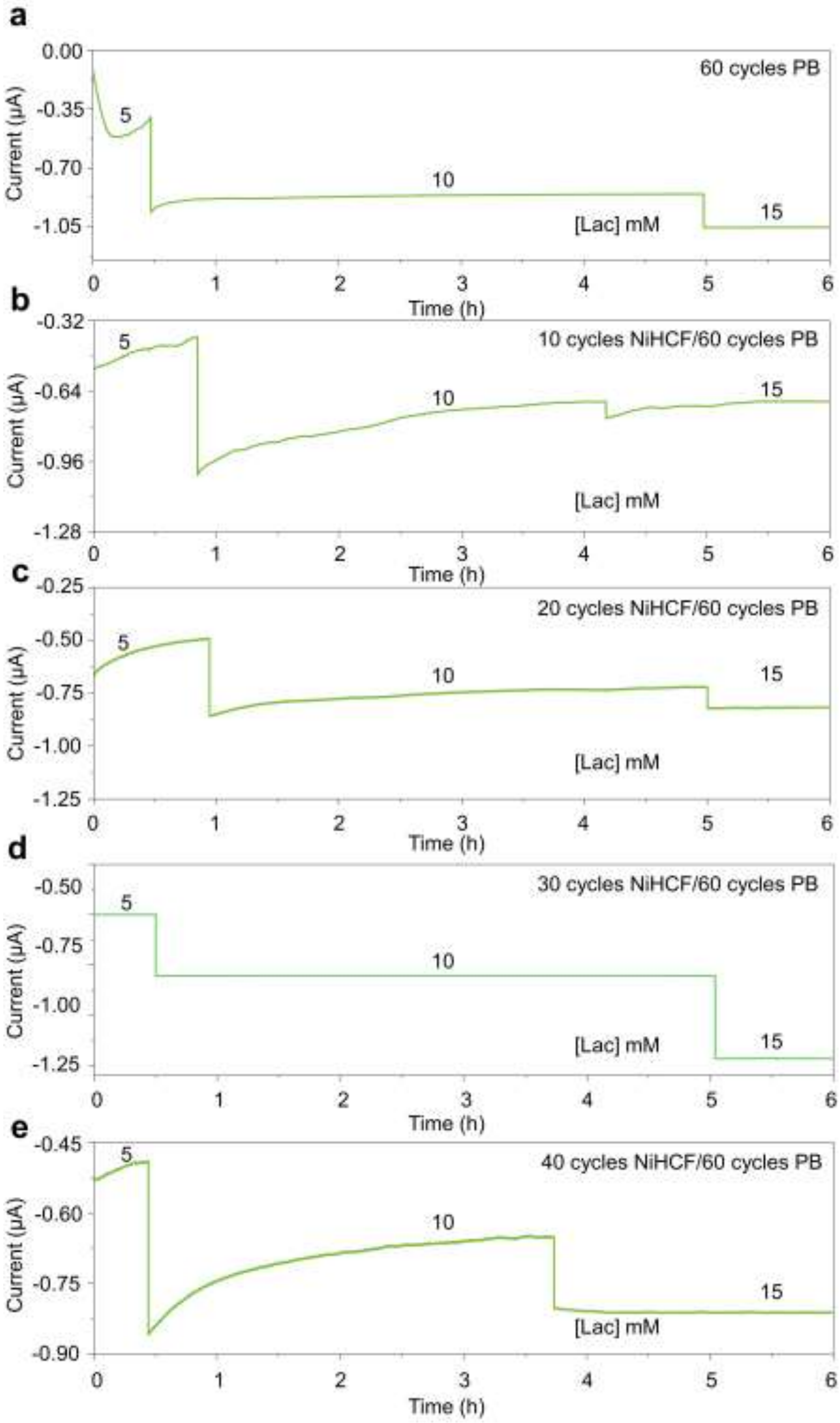

Figure S24. The long-term stability test for lactate biosensing fibers prepared with varying NiHCF deposition cycles. Amperometric responses of lactate biosensing fibers based on PB (a), PB with 10 cycles of NiHCF (b), PB with 20 cycles of NiHCF (c), PB with 30 cycles of NiHCF (d), and PB with 40 cycles of NiHCF (e) in PBS solutions containing 5, 10, 15 mM lactate.

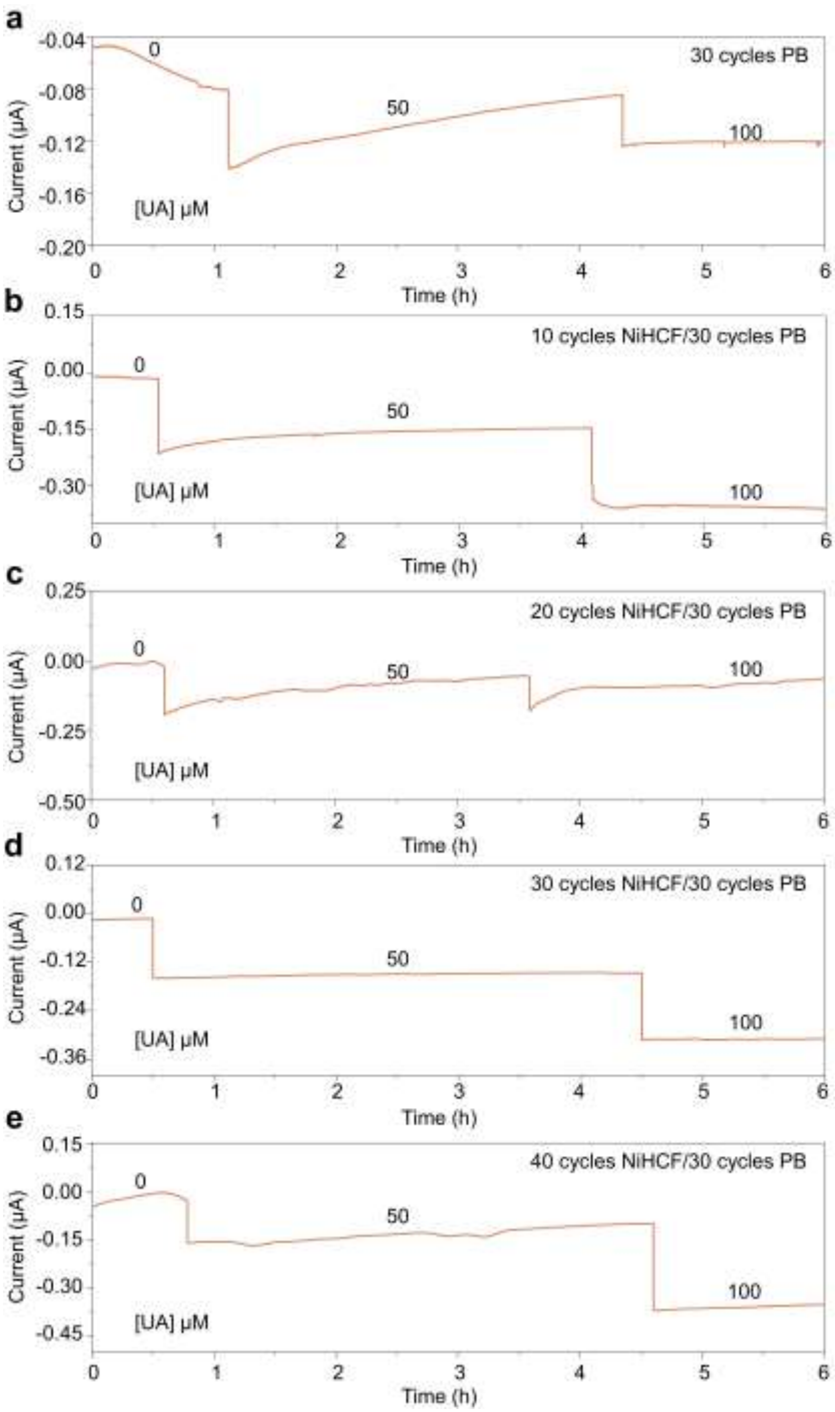

Figure S25. The long-term stability test for uric acid biosensing fibers prepared with varying NiHCF deposition cycles. Amperometric responses of uric acid biosensing fibers based on PB (a), PB with 10 cycles of NiHCF (b), PB with 20 cycles of NiHCF (c), PB with 30 cycles of NiHCF (d), and PB with 40 cycles of NiHCF (e) in PBS solutions containing 0, 50, 100 μM uric acid.

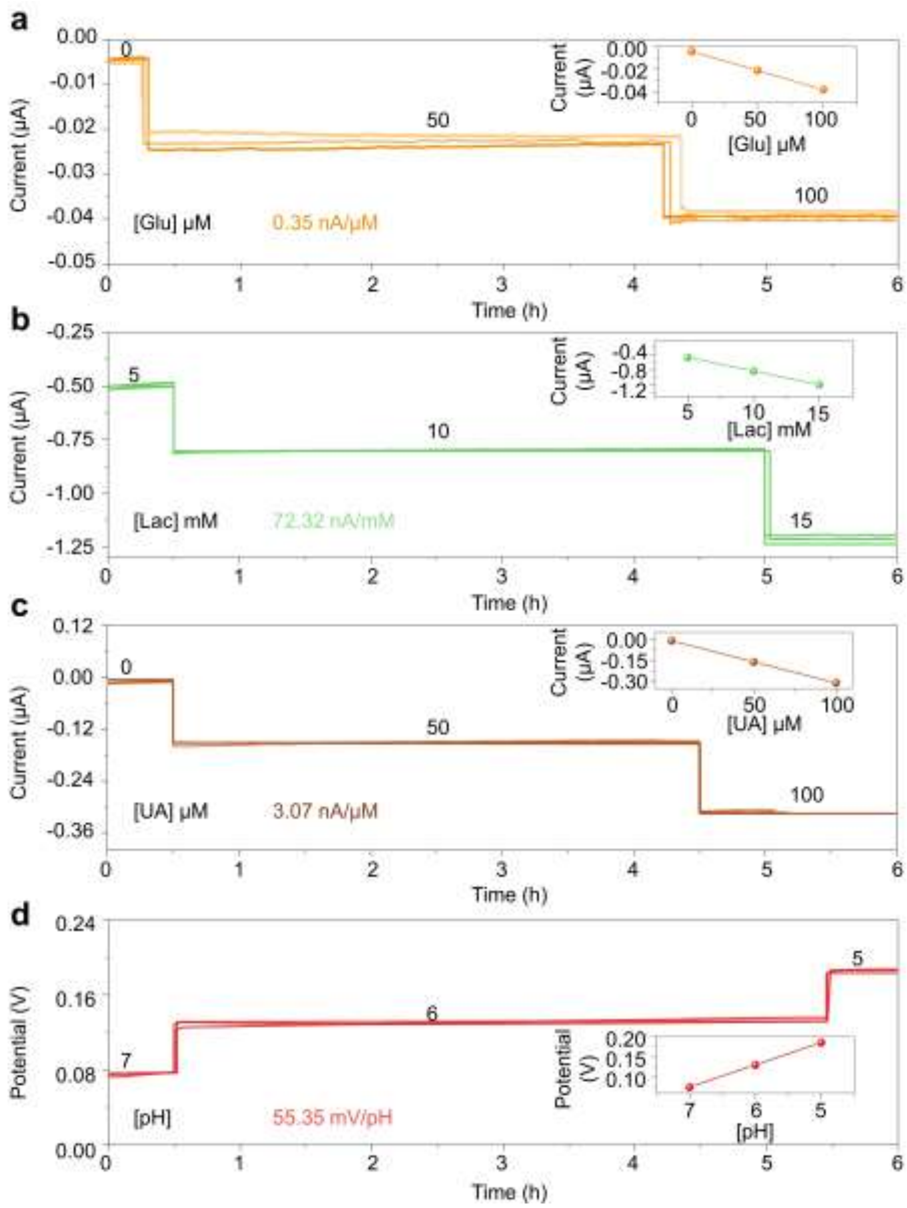

Figure S26. Reproducibility of the long-term stability of the glucose (a), lactate (b), uric acid (c), and pH (d) biosensing fibers (n=3), with relative standard deviations of 13.04%, 1.84%, 1.32%, and 1.20%, respectively.

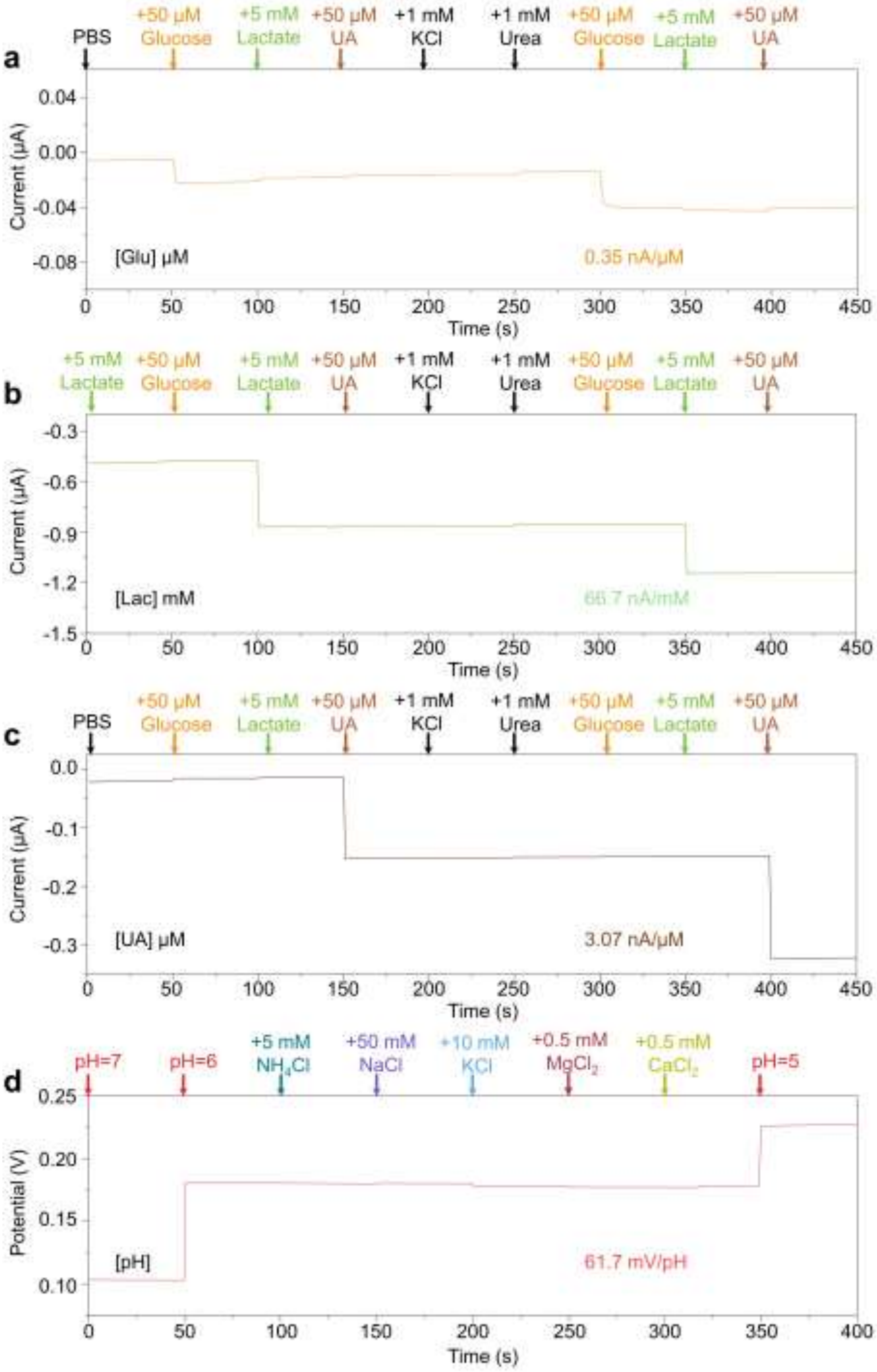

Figure S27. Selectivity of glucose (a), lactate (b), uric acid (c), and pH (d) biosensing fibers. Physiologically pertinent levels of common sweat constituents were introduced into a standard solution.

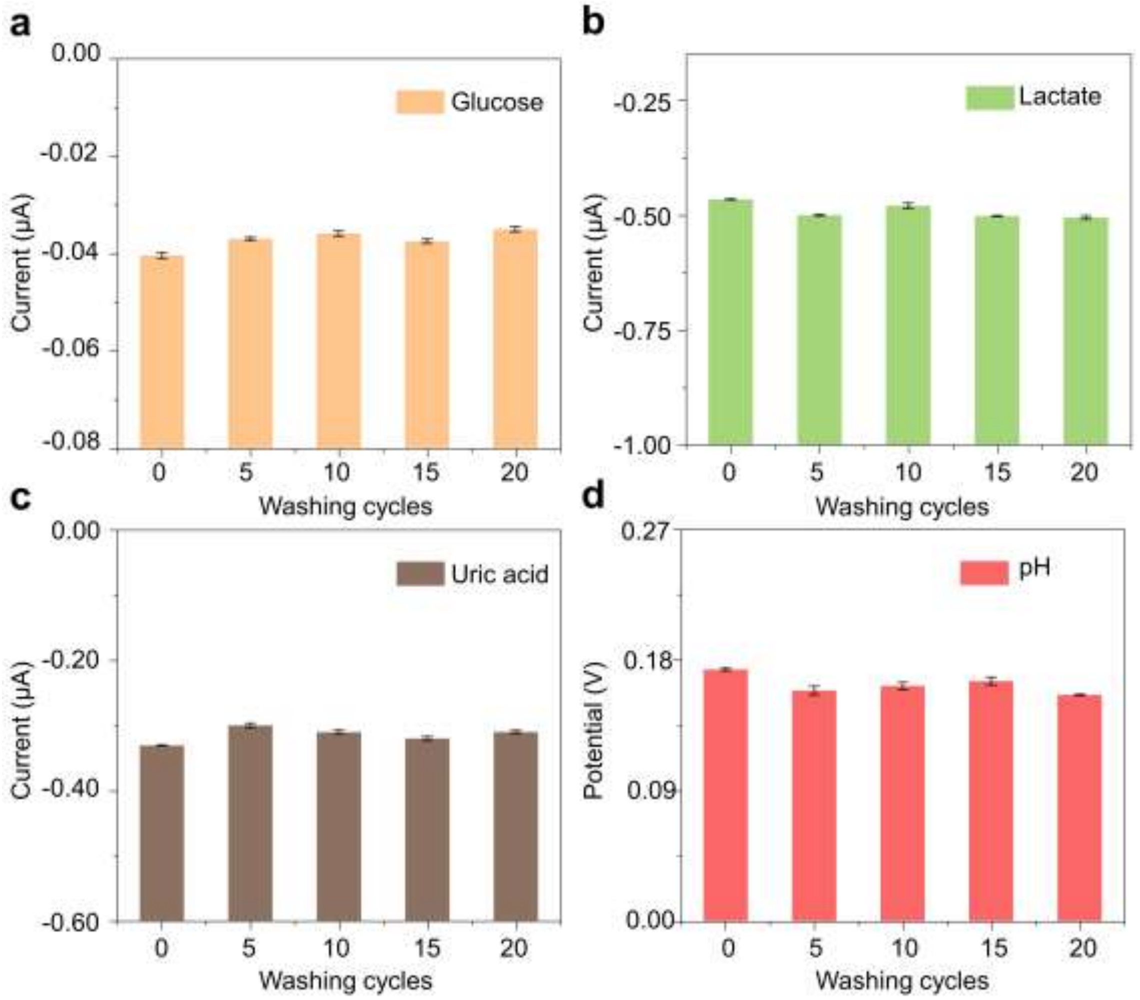

Figure S28. The washability of the electrochemical fabric including glucose (a), lactate (b), uric acid (c), and pH (d) biosensing fibers under repeated water washing (n=3, mean ± s.d).

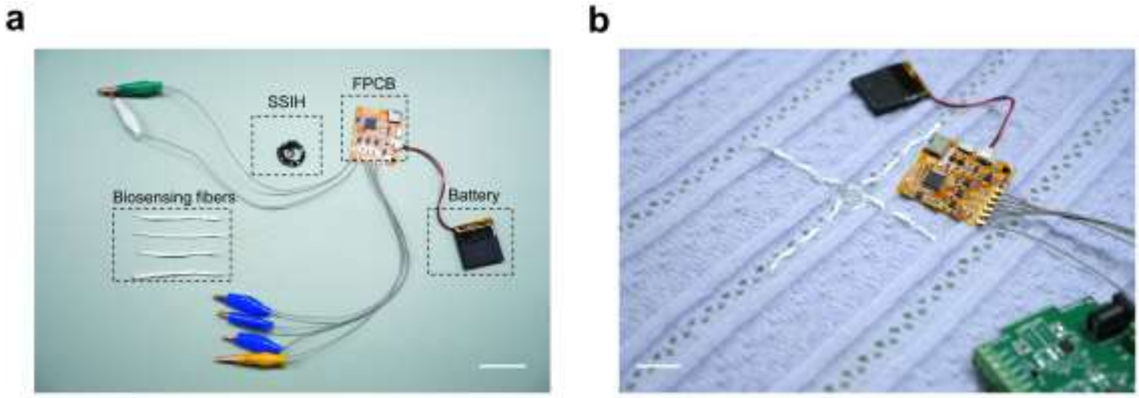

Figure S29. (a) Photograph showing the components of the electrochemical fabric biosensing system, including the biosensing fibers, ionophoresis hydrogel, FPCB, and battery modules. Scale bar, 3 cm. (b) Photograph showing the integration of these components into everyday clothes. Scale bar, 1.5 cm.

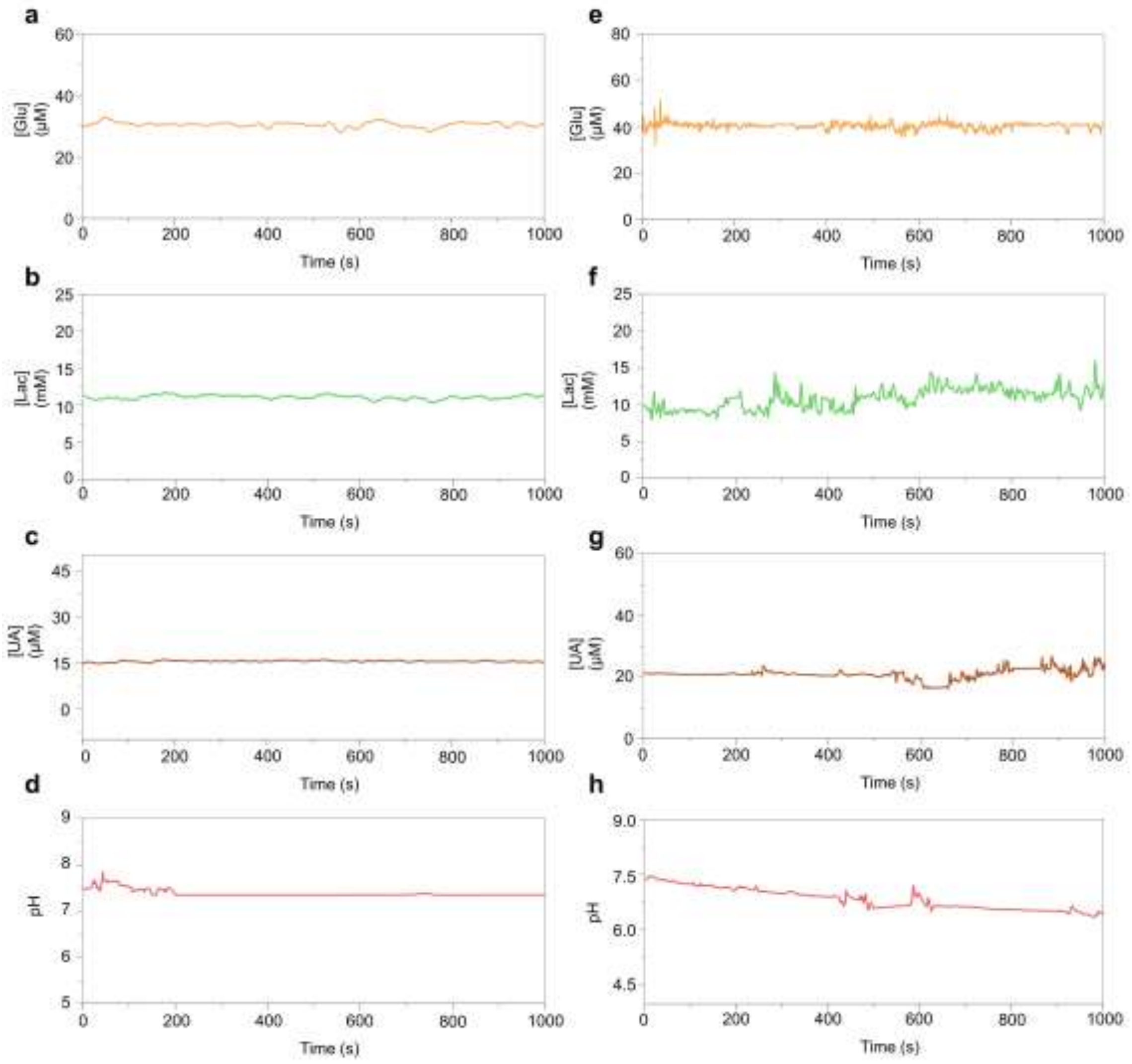

Figure S30. Comparison of output signal stability in on-body evaluation: (a-d) sedentary state and (e-h) running.

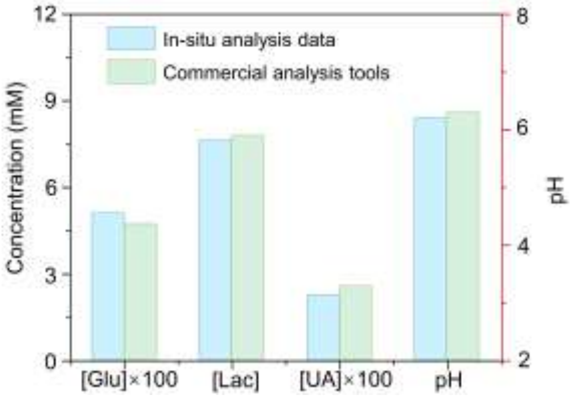

Figure S31. Comparison of in-situ multiplexed biomarkers data with ex-situ data from the collected sweat samples via commercial tools (LC-MS/MS and pH meter).

Table S1. List of operational parameters for reported hydrogel-mediated iontophoretic transdermal healthcare systems

Application	Drug	Hydrogel type	Biomarkers	Operational current (μA)	Current density ($mA\,cm^{-2}$)	Ref.
Wound management	-	PVA	-	8000	2.00	[2]
	-	PVA	-	8000	15.92	[3]
	-	PVA	-	300	4.25	[4]
	[VBIM]Br	PVA	-	250	0.05	[5]
Drug delivery	Insulin	Polyacrylamide	-	4000	4.00	[6]
	Insulin	Agarose	-	1000	0.06	[7]
	Sulforhodamine B	P(AM-co-SV)	-	1125	0.18	[8]
	Dextran	Agarose	-	3000	3.00	[9]
Sweat induction	Pilocarpine	PVA	Glu, Alc	800	0.30	[10]
	Acetylcholine	Agarose	Na^+,Cl^-, Glu	1000	0.07	[11]
	Pilocarpine	PVA	Alc	600	0.20	[12]
	Carbachol	Agarose	pH, Ca^{2+}, K^+, Na^+, Glu	200	0.05	[13]
	Pilocarpine	Agarose	Glu, Lac, Alc, Caf	900	0.30	[14]
	Carbachol	Agarose	Amino acids, Vitamin	100	0.26	[15]
	Pilocarpine	Agarose	Cl^-, zinc, iron	250	0.50	[16]
	Pilocarpine	PVA	Glu	1000	1.00	[17]
	Pilocarpine	Agarose	Glu, Lac	200	0.30	[18]
	Carbachol	Agarose	β-hydroxybutyrate	150	0.40	[19]
	**Carbachol**	**PVA-PAA**	**pH, Glu, Lac, UA**	**75**	**0.032**	**This work**

PVA, polyvinyl alcohol; P(AM-co-SV), poly(acrylamide-co-*p*-styrene-bipyridine); Alc, alcohol; Glu, glucose; Lac, lactate; Caf, caffeine; UA, uric acid; PAA, poly(acrylic acid).

Table S2. Summary of healthy human subjects demographics.

Subject	Gender	Age (years)	BMI (kg/m^2)
1	Female	25	19.23
2	Male	23	21.79
3	Female	25	20.75
4	Male	35	18.08
5	Male	30	27.76

References:

1. Li Y-L, Kinloch IA, Windle AH. Direct spinning of carbon nanotube fibers from chemical vapor deposition synthesis. *Science* 2004; **304**: 276-8.

2. Lei H, Fan D. Conductive, adaptive, multifunctional hydrogel combined with electrical stimulation for deep wound repair. *Chem Eng J* 2021; **421**: 129578.

3. Zheng X, Yao J, Yao J *et al.* Multifunctional hydrogel combined with electrical stimulation therapy for promoting diabetic wound healing. *Nano Res* 2024; **17**: 9942-53.

4. Wang K, Parekh U, Ting JK *et al.* A platform to study the effects of electrical stimulation on immune cell activation during wound healing. *Adv Biosyst* 2019; **3**: 1900106.

5. Liu B, Fu R, Duan Z *et al.* Ionic liquid-based non-releasing antibacterial, anti-inflammatory, high-transparency hydrogel coupled with electrical stimulation for infected diabetic wound healing. *Composites, Part B* 2022; **236**: 109804.

6. Wang H, Cai R, Wang S *et al.* A wearable transdermal device for on-demand drug delivery. *Matter* 2025; **8**: 102040.

7. Li Y, Yang J, Zheng Y *et al.* Iontophoresis-driven porous microneedle array patch for active transdermal drug delivery. *Acta Biomater* 2021; **121**: 349-58.

8. Zhou Y, Jia X, Pang D *et al.* An integrated Mg battery-powered iontophoresis patch for efficient and controllable transdermal drug delivery. *Nat Commun* 2023; **14**: 297.

9. Kusama S, Sato K, Matsui Y *et al.* Transdermal electroosmotic flow generated by a porous microneedle array patch. *Nat Commun* 2021; **12**: 658.

10. Kim J, Sempionatto JR, Imani S *et al.* Simultaneous Monitoring of Sweat and Interstitial Fluid Using a Single Wearable Biosensor Platform. *Adv Sci* 2018; **5**: 1800880.

11. Emaminejad S, Gao W, Wu E *et al.* Autonomous sweat extraction and analysis applied to cystic fibrosis and glucose monitoring using a fully integrated wearable platform. *Proc Natl Acad Sci* 2017; **114**: 4625-30.

12. Kim J, Jeerapan I, Imani S *et al.* Noninvasive alcohol monitoring using a wearable tattoo-based iontophoretic-biosensing system. *ACS Sens* 2016; **1**: 1011-19.

13. Peringeth K, Ganguly A, Pal A *et al.* Self-powered microfluidic-based sensor for noninvasive sweat analysis. *Sens. Actuators, B* 2025; **423**: 136859.
14. Sempionatto JR, Lin M, Yin L *et al.* An epidermal patch for the simultaneous monitoring of haemodynamic and metabolic biomarkers. *Nat Biomed Eng* 2021; **5**: 737-48.
15. Wang M, Yang Y, Min J *et al.* A wearable electrochemical biosensor for the monitoring of metabolites and nutrients. *Nat Biomed Eng* 2022; **6**: 1225-35.
16. Kim J, Oh S, Yang DS *et al.* A skin-interfaced, miniaturized platform for triggered induction, capture and colorimetric multicomponent analysis of microliter volumes of sweat. *Biosens Bioelectron* 2024; **253**: 116166.
17. Zhao J, Lin Y, Wu J *et al.* A fully integrated and self-powered smartwatch for continuous sweat glucose monitoring. *ACS Sens* 2019; **4**: 1925-33.
18. Hojaiji H, Zhao Y, Gong MC *et al.* An autonomous wearable system for diurnal sweat biomarker data acquisition. *Lab Chip* 2020; **20**: 4582-91.
19. Shen Y, Chen G, Chen Y *et al.* Wearable microfluidic electrochemical sensor integrated with iontophoresis for non-invasive sweat ketone monitoring. *Sens. Actuators, B* 2024; **421**: 136518.
